# RavN is a member of a previously unrecognized group of *Legionella pneumophila* E3 ubiquitin ligases

**DOI:** 10.1371/journal.ppat.1006897

**Published:** 2018-02-07

**Authors:** Yi-Han Lin, María Lucas, Timothy R. Evans, Guillermo Abascal-Palacios, Alexandra G. Doms, Nicole A. Beauchene, Adriana L. Rojas, Aitor Hierro, Matthias P. Machner

**Affiliations:** 1 Division of Molecular and Cellular Biology, *Eunice Kennedy Shriver* National Institute of Child Health and Human Development, National Institutes of Health, Bethesda, Maryland, United States of America; 2 Structural Biology Unit, CIC bioGUNE, Bizkaia Technology Park, Derio, Spain; 3 IKERBASQUE, Basque Foundation for Science, Bilbao, Spain; Gifu University, JAPAN

## Abstract

The eukaryotic ubiquitylation machinery catalyzes the covalent attachment of the small protein modifier ubiquitin to cellular target proteins in order to alter their fate. Microbial pathogens exploit this post-translational modification process by encoding molecular mimics of E3 ubiquitin ligases, eukaryotic enzymes that catalyze the final step in the ubiquitylation cascade. Here, we show that the *Legionella pneumophila* effector protein RavN belongs to a growing class of bacterial proteins that mimic host cell E3 ligases to exploit the ubiquitylation pathway. The E3 ligase activity of RavN was located within its N-terminal region and was dependent upon interaction with a defined subset of E2 ubiquitin-conjugating enzymes. The crystal structure of the N-terminal region of RavN revealed a U-box-like motif that was only remotely similar to other U-box domains, indicating that RavN is an E3 ligase relic that has undergone significant evolutionary alteration. Substitution of residues within the predicted E2 binding interface rendered RavN inactive, indicating that, despite significant structural changes, the mode of E2 recognition has remained conserved. Using hidden Markov model-based secondary structure analyses, we identified and experimentally validated four additional *L*. *pneumophila* effectors that were not previously recognized to possess E3 ligase activity, including Lpg2452/SdcB, a new paralog of SidC. Our study provides strong evidence that *L*. *pneumophila* is dedicating a considerable fraction of its effector arsenal to the manipulation of the host ubiquitylation pathway.

## Author summary

Bacterial pathogens often hijack conserved host pathways by encoding proteins that are molecular mimics of eukaryotic enzymes, thus tricking the host cell into surrendering its resources to the bacteria. Here, we show that the intracellular pathogen *Legionella pneumophila* uses such a strategy to exploit ubiquitylation, a conserved post-translational modification that is mediated by E3 ubiquitin ligases. *L*. *pneumophila* encodes molecular mimics of host E3 ligases, including the effector protein RavN, thereby subverting the ubiquitylation pathway for its own benefit during infection. Using protein crystallography, we show that the fold of RavN has only residual resemblance to conventional eukaryotic E3s, yet its mode of interaction with E2 enzymes, host proteins that are important for the ubiquitin transfer reaction, has been preserved throughout evolution. Inspired by the discovery of RavN, we performed an *in silico* fold homology search and discovered several additional E3 ligase candidates within the effector repertoire of *L*. *pneumophila* that, until now, had remained hidden due to lack of primary sequence similarity. Our study supports the hypothesis that E3 ligases are a vital part of the virulence program of *L*. *pneumophila*, and that these effectors, despite having undergone extensive evolutionary changes, have retained features that are critical for their biological function, including the ability to hijack host factors that are part of the ubiquitylation machinery.

## Introduction

Ubiquitylation is one of the most significant and versatile post-translational modifications in cell biology. It shapes almost all cellular processes including protein homeostasis and trafficking, signal transduction, and cell cycle progression [1–3]. The hallmark of ubiquitylation is the covalent attachment of the 76-amino acid protein ubiquitin (Ub) to substrate proteins, a process that requires the sequential action of three classes of enzymes: Ub-activating enzymes (E1s), Ub-conjugating enzymes (E2s), and Ub ligases (E3s). The E1 forms a thioester bond with Ub in an adenosine triphosphate (ATP)-dependent manner and transfers Ub onto an active site cysteine (Cys) residue within the E2. From there, Ub is transferred onto target proteins by E3 ligases which facilitate the formation of an isopeptide bond between the C-terminal glycine (Gly) residue of Ub and, in most cases, the ε-amine of lysine (Lys) residues within a target protein. The primary sequence of Ub contains seven Lys residues (at position 6, 11, 27, 29, 33, 48, and 63), and poly-ubiquitin chains can be formed via different Lys linkages. The type of linkage ultimately determines the fate of the substrate protein [4, 5]. For example, proteins with Lys48-linked poly-ubiquitin chains are marked for degradation by the 26S proteasome [3], whereas Lys63-linkages regulate endocytosis and protein localization [6–8]. As is true for most post-translational modifications, ubiquitylation can be reversed by specific peptidases called deubiquitylating enzymes that remove individual ubiquitin molecules or entire ubiquitin chains from substrate proteins [9].

E3 ubiquitin ligases play a central role in the ubiquitylation system due to their ability to simultaneously recognize the substrate protein and the E2-ubiquitin conjugate. Over 500 eukaryotic E3s have been identified [10] and are categorized into two major classes based on their fold and catalytic mechanism [11, 12]: HECT (Homologous to the E6-AP Carboxyl Terminus)-type E3s form a transient intermediate with ubiquitin via a Cys residue in their active site prior to ubiquitin transfer onto the substrate protein, while RING (Really Interesting New Gene) or U-box-type E3s function as a scaffold by bringing the E2-ubiquitin conjugate into close proximity with the target protein in order for Ub transfer to occur, notably without the formation of a covalent E3-Ub intermediate. Given the importance of ubiquitylation in eukaryotic cell physiology and protein regulation, it is not surprising that this post-translational modification is exploited by a variety of pathogens that encode molecular mimics of E3 ligases [13–15]. For example, the *Salmonella* effector protein SopA is a HECT-type E3 ligase that modulates the proinflammatory response through ubiquitylation [16, 17]. Similarly, *Pseudomonas* AvrPtoB, a U-box type E3 ligase, ubiquitylates host kinases to suppress the plant innate immunity [18–20]. In addition to using molecular mimics of host E3s, bacteria have also evolved novel types of E3 ligases that, despite the lack of fold-similarity to eukaryotic proteins, rely on E2s to mediate ubiquitylation. These NELs (Novel E3 Ligases) include the effectors SspH1 and SspH2 from *Salmonella* [21], NopM from *Rhizobium* [22], and several IpaH protein family members from *Shigella* [23, 24]. Thus, pathogens have acquired or developed E3 ligases in a variety of ways, underscoring the importance of ubiquitylation for microbial virulence.

*Legionella pneumophila* is a facultative intracellular pathogen that replicates within amoeba in the environment and within alveolar macrophages during Legionnaires’ disease, a potentially fatal pneumonia [25]. This Gram-negative bacterium translocates close to 300 effector proteins into the infected host cell. Only a fraction of these effectors has been characterized in detail [26], while the majority of them are of unknown function due to the lack of primary sequence homology to other entries in the database. Until now, a total of five *L*. *pneumophila* effector proteins have been experimentally confirmed to exploit host cell ubiquitylation by altering or mimicking E3 ligase activity: LegAU13/AnkB and LegU1 contain F-box domains, a 50 amino acid domain that interacts with the SCF (Skp, Cullin, F-box containing) complex for substrate protein ubiquitylation [27–29], whereas LubX and GobX are U-box domain-containing E3 ligases [30–32]. Although the identity of the target of GobX remains to be determined, the fact that it exploits host-mediated S-acylation (palmitoylation) in order to localize to Golgi membranes suggests that it functions at or in close proximity to this compartment [32]. Unlike GobX, LubX possesses two U-box domains and mediates poly-ubiquitylation of host protein Clk1 (cdc2-like kinase 1) [30]. In addition, LubX is a meta-effector that ubiquitylates another *L*. *pneumophila* effector, SidH, and targets it for degradation [33]. The effector SidC enhances endoplasmic reticulum (ER) recruitment to the *Legionella*-containing vacuole (LCV) [34] and mediates the formation of poly-ubiquitin conjugates on the LCV [35]. The E3 ligase fold of SidC and its paralog SdcA differs from that of known E3 ligases, including the NEL family, and represents yet another family of bacterial E3 ligases [35]. More recently, members of the SidE family of effector proteins were shown to promote ubiquitylation, notably in an E1- and E2-independent manner. The mono-ADP-ribosyltransferase (mART) domain of SidE uses nicotinamide adenine dinucleotide (NAD), instead of ATP, to activate ubiquitin via an ADP-ribosylated ubiquitin intermediate [36]. The phosphodiester bond in the ADP-ribosylated ubiquitin is then cleaved by the nucleotidase-phosphohydrolase (NP) domain of SidE, which results in the formation of the phospho-ribosylated ubiquitin that subsequently attaches to the substrate protein [37, 38]. Undoubtedly, the structural and mechanistic variety among *L*. *pneumophila* effectors that exploit the host ubiquitylation machinery suggests an important role for ubiquitylation for a successful infection, yet the true depth of this host-pathogen interplay remains to be determined.

Using a combinatorial approach of protein biochemistry, structural biology, as well as *in silico* studies, we discovered and characterized a previously unrecognized group of *L*. *pneumophila* effectors that exhibit E3 ubiquitin ligase activity, which suggests that *L*. *pneumophila* exploits host cell ubiquitylation to an extent larger than previously expected.

## Results

### The *Legionella* effector RavN is a bona fide E3 ligase

**Fig 1:**
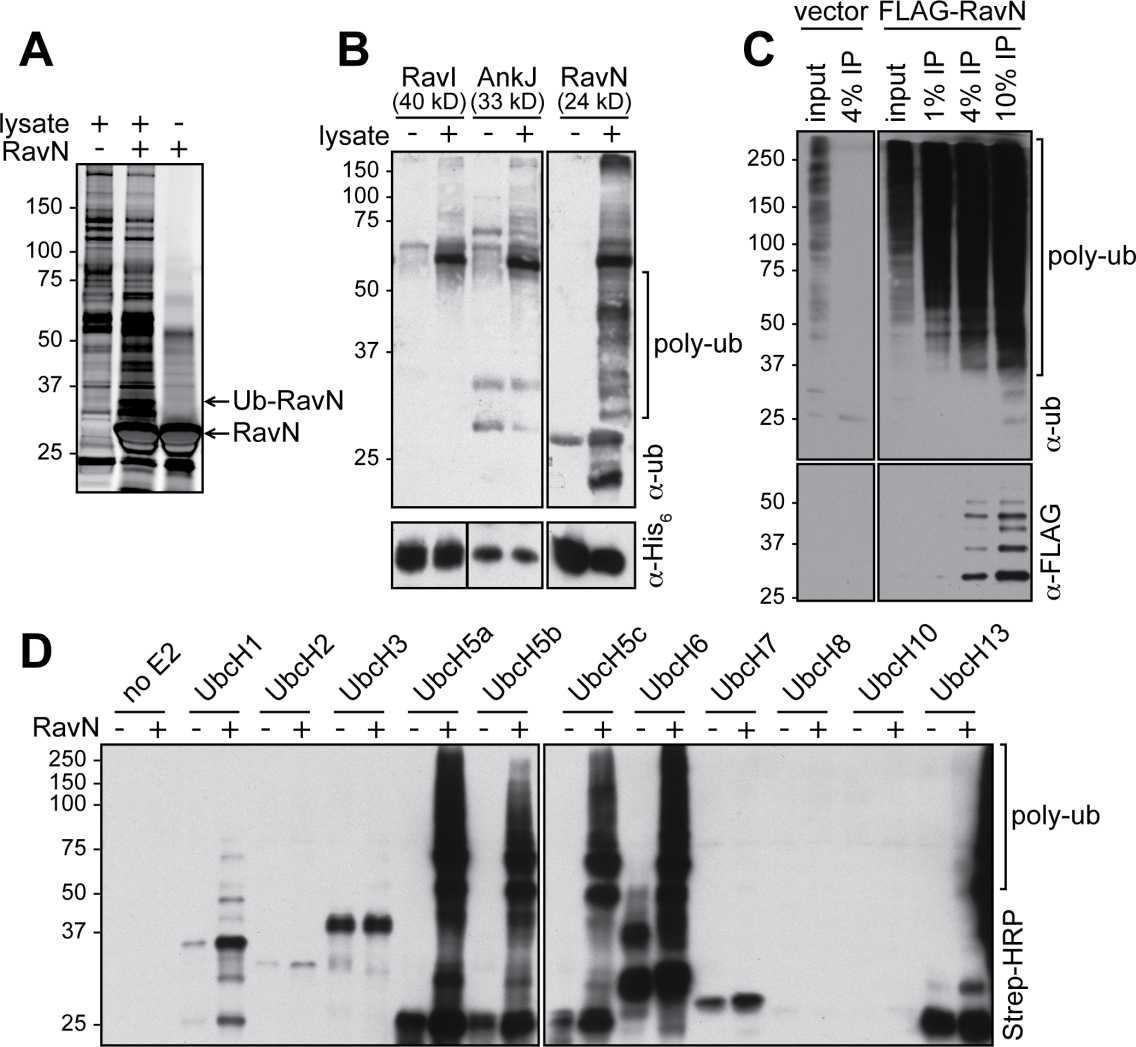
RavN has ubiquitin ligase activity. (A) The silver-stained SDS-PAGE gel showing proteins present on either uncoated control beads (left) or His_6_-RavN-coated beads before (right) or after (center) incubation with lysate from HEK293T cells. The protein bands corresponding to His_6_-RavN or mono-ubiquitylated His_6_-RavN are indicated with arrows. (B) His_6_-tagged RavN, but not RavI or AnkJ, is ubiquitylated upon incubation with HEK293T cell lysate. Top: Poly-ubiquitylated species (poly-ub) detected by immunoblot using anti-ubiquitin antibody. Bottom: Total amount of His_6_-tagged effector proteins present in each lane. (C) FLAG-RavN is poly-ubiquitylated in transiently transfected HEK293T cells. FLAG-tagged RavN was precipitated from HEK293T cell lysate, and ubiquitylation (poly-ub) was detected by immunoblot using anti-ubiquitin antibody. Total amount of FLAG-RavN was detected by anti-FLAG antibody shown at the bottom. (D) *In vitro* ubiquitylation assay. Purified recombinant GST-RavN was incubated in the presence of a panel of mammalian E2s or without E2s (no E2), and the formation of poly-ubiquitylated species (poly-ub) was detected using HRP-conjugated streptavidin.

The functional characterization of *L*. *pneumophila* effector-encoding genes has been complicated by the fact that their deletion, individually or even in groups, rarely results in a detectable phenotypic effect [39]. Consequently, standard genetic approaches have been mostly unsuccessful in deciphering this functional redundancy among effectors. We therefore chose a discovery-based method of first identifying direct host interaction partners for *L*. *pneumophila* effectors and then characterizing these novel host-pathogen interactions in detail. Lysate from human embryonic kidney (HEK293T) cells was incubated with agarose beads coated with a panel of hexahistidine (His_6_)-tagged *L*. *pneumophila* effectors. Host proteins retained by the beads were eluted, separated by sodium dodecyl sulfate-polyacrylamide gel electrophoresis (SDS-PAGE), and visualized by silver staining. When using RavN (Lpg1111; 212 amino acids, 24 kDa), a translocated substrate of the Dot/Icm Type IV secretion system (T4SS) [40, 41], as bait, a protein of approximately 35 kDa in size was highly enriched in the pulldown fraction but not in the control lanes (Fig 1A). Upon tryptic digestion and high-performance liquid chromatography/mass spectrometry (HPLC/MS) analysis, the protein in the 35 kDa band gel slice was identified as the mono-ubiquitylated form of His_6_-RavN. Importantly, other *L*. *pneumophila* effector proteins, such as His_6_-RavI (~40.4 kDa) or His_6_-AnkJ (~32.9 kDa), showed only weak ubiquitylation under similar experimental conditions (Fig 1B), suggesting that the ubiquitin modification was preferentially attached to RavN.

Next, we analyzed if the post-translational modification of RavN with ubiquitin also occurred in the context of living cells. Upon immuno-precipitation of FLAG-tagged RavN from lysate of transiently transfected HEK293T cells (Fig 1C), we detected robust poly-ubiquitylation in lanes containing FLAG-RavN but not in the control lane (vector only). Together, these studies confirmed that RavN was modified with ubiquitin upon exposure to cytosolic content of eukaryotic cells.

The post-translational modification of RavN with ubiquitin upon exposure to human cells or cell lysate could be explained in a variety of ways. One possibility was that RavN was randomly targeted by one or several eukaryotic E3 ubiquitin ligases. This scenario seemed unlikely given the fact that efficient ubiquitylation of RavN was detectable even under enzymatically unfavorable conditions–namely incubation at low temperatures (4°C) and with a molar excess of RavN relative to host E3 ligases within the lysate (Fig 1A). Moreover, none of the other *L*. *pneumophila* effectors tested here were post-translationally modified with ubiquitin under similar experimental conditions (Fig 1B). Hence, we favored an alternative possibility in which RavN functioned as a bona fide E3 ubiquitin ligase and had auto-ubiquitylated, a phenomenon common among E3 ligases [42, 43]. To test this hypothesis, we performed an *in vitro* reconstitution assay in which purified glutathione S-transferase (GST)-tagged RavN was incubated with the minimal components necessary for protein ubiquitylation to occur, including an E1, a panel of eleven different mammalian E2s, ATP, and biotinylated ubiquitin. The ability of GST-RavN to catalyze poly-ubiquitylation was then assessed by SDS-PAGE and immunoblot using HRP-conjugated streptavidin (Fig 1D). While control reactions lacking either GST-RavN or an E2 underwent no noticeable poly-ubiquitylation, efficient poly-ubiquitylation was detected upon incubation of GST-RavN with the E2 enzymes UbcH5a, UbcH5b, UbcH5c, and UbcH6, but none of the other E2s, indicating that RavN was indeed an E3 ubiquitin ligase that preferred a select set of host E2s.

### The E3 ligase activity of RavN is located within the N-terminal region

**Fig 2:**
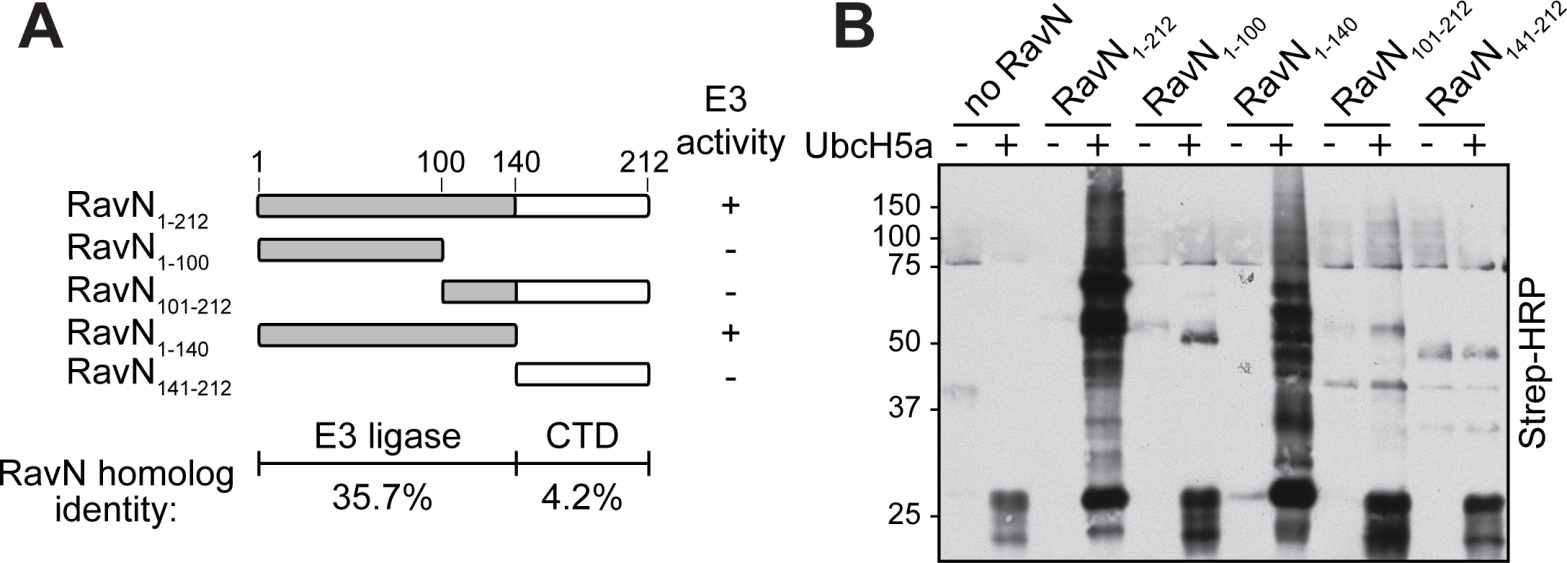
The N-terminal region of RavN mediates E3 ligase activity. (A) Shown here is the schematic representation of the RavN fragments examined in the *in vitro* ubiquitylation assay in (B). The degree of sequence identity of the N-terminal E3 ligase domain and C-terminal region (CTD) among *Legionella* RavN homologs (as seen in S2A Fig) is indicated [in %]. (B) The *in vitro* ubiquitylation assay of RavN truncation variants was performed as described in Fig 1D using UbcH5a as E2. The formation of poly-ubiquitylated species was detected with HRP-conjugated streptavidin.

Our Basic Local Alignment Search Tool (BLAST) analysis using the primary sequence of RavN as query revealed no homology to known E3 ligases. Thus, to determine which region of RavN was responsible for its E3 ligase activity, we generated truncated variants of GST-RavN and analyzed their *in vitro* ubiquitylation activity upon incubation with UbcH5a (Fig 2), one of the preferred E2s of full-length RavN (Fig 1D). While none of the variants with N-terminal truncations (RavN_101-212_, RavN_141-212_) showed any detectable E3 ligase activity, a variant comprised of amino acid residues 1–140 (RavN_1-140_) exhibited E3 ligase activity similar to that of full-length RavN (Fig 2). Further truncation of RavN_1-140_ from its C-terminal end (resulting in RavN_1-100_) abolished E3 ligase activity, indicative of the E3 ligase domain of RavN being located within the N-terminal 140 amino acid residues.

A recent genome analysis revealed a high level of heterogeneity in the repertoire of effectors among 41 different *Legionella* species [44]. Using BLAST, we discovered that RavN homologs are present in most sequenced *L*. *pneumophila* isolates (Paris, Lens, Corby, Alcoy, Wadsworth, Thunder Bay) and in nine other *Legionella* species (*Legionella bozemanae* [now *Fluoribacter bozemanae*], *L*. *tucsonensis*, *L*. *anisa*, *L*. *parisiensis*, *L*. *gratiana*, *L*. *santicrucis*, *L*. *cincinnatiensis*, *L*. *longbeachae*, and *L*. *sainthelensi*) that belong to two closely related branches of the same clade in the *Legionella* phylogenetic tree (S1A Fig) [44]. Upon sequence alignment of the various RavN homologs, we found that the N-terminal 140 residues, the region that mediated E3 activity, showed a notably higher level of conservation (35.7% identity) than the C-terminal region (residues 141–212; 4.2% identity) of unknown function (Fig 2A and S2A Fig). This result further suggested that the E3 ligase domain of RavN is important for its biological function and has, accordingly, been maintained throughout evolution in homologs from various *Legionella* species and isolates. During *L*. *pneumophila* growth in liquid media, the production levels of RavN were highest during late exponential/stationary phase (S1B Fig), a phase that is equivalent to the transmissive (= virulent) intracellular form. This finding suggested that RavN is most likely translocated by *L*. *pneumophila* during the early stage of infection. However, deletion of *ravN* from the *L*. *pneumophila* genome did not result in a detectable growth defect within U937 macrophages (S1C Fig), suggesting that RavN is not essential for survival within human host cells and that other functionally redundant effectors might compensate for the loss of RavN.

Although the identity of the host target(s) of RavN has yet to be determined, we analyzed the subcellular localization of this newly discovered E3 ligase in order to gain first insight into its putative site(s) of function. Using enhanced monomeric green fluorescent protein (EmGFP) as reporter, we discovered that RavN was distributed throughout the cytosol of transiently transfected COS-1 cells (S1D Fig). To confirm these results, we performed membrane fractionation assays on human U937 macrophages that had been challenged with *L*. *pneumophila* (S1E Fig). Upon translocation into U937 cells, RavN was almost exclusively found in the cytosolic fraction together with the marker protein glyceraldehyde 3-phosphate dehydrogenase (GAPDH), whereas only little RavN was present in the membrane-containing fraction that was enriched for the marker protein calnexin (S1E Fig). Thus, the putative target of RavN was most likely not a membrane-associated host factor.

### The E3 ligase domain of RavN contains a U-box-like fold

Given the aforementioned lack of primary sequence similarity between RavN and known E3 ligases, we turned to structural biology in order to gain insights into the molecular basis of the ubiquitylation activity of RavN. Initial attempts to crystallize full-length RavN were unsuccessful. Thus, we carried out limited trypsin-mediated proteolysis in combination with Matrix Assisted Laser Desorption/Ionization–Time of Flight (MALDI–TOF) mass spectrometry to identify stable fragments that may be more amenable to crystallization (as described in Materials and Methods). This approach proved successful in producing a RavN fragment (residues 1–123; RavN_1-123_) that existed as a monomer in solution (S3A Fig) and that crystallized readily. The structure was solved by single isomorphous replacement with anomalous signal (SIRAS) using iodine-soaked crystals. The calculated electron density map allowed unambiguous tracing of the complete RavN_1-123_ amino acid sequence (S3B and S3C Fig). The final model was refined to 1.8Å resolution with R_work_ / R_free_ values of 15 / 19% and excellent stereochemistry. Data collection, phasing and refinement statistics are summarized in Table 1.

**Table 1:** Crystallographic data collection, phasing and refinement statistics.

	Native	Iodide-SAD
**Data collection**		
Space group	C222	C222
Cell dimensions		
*a*, *b*, *c* (Å)	114.2, 177.0, 79.4	114.6, 177.5, 80.4
*α*, *β*, *γ* (°)	90, 90, 90	90, 90, 90
Resolution (Å)	48.0–1.72 (1.82–1.72)*	57.3–2.39 (2.54–2.39)
*R*_meas_	0.06 (0.91)	0.11 (0.85)
CC_1/2_	0.99 (0.72)	0.997 (0.711)
*I*/σ*I*	24.6 (3.1)	12.5 (2.3)
Completeness (%)	99.7 (98.6)	99.7 (98.0)
Multiplicity	13.2 (12.7)	12.4 (11.2)
Anomalous completeness (%)		99.2 (95.0)
Anomalous multiplicity		6.6 (6.0)
Wavelength	0.97887	1.90748
**Refinement**		
Resolution (Å)	48.0–1.872	
No. reflections	85742	
*R*_work/_ *R*_free_	0.15/0.19	
No. of non-H atoms		
Protein	3089	
Ligand**	83	
Water	271	
B-factors		
Protein	48.2	
Ligand	70.2	
Water	61.3	
R.m.s deviations		
Bond lengths (Å)	0.011	
Bond angles (°)	1.09	
**PDB code**	5MIY	

* Values in parentheses correspond to the highest resolution shell.

** Ligands present in the structures are: ethylene glycol {(CH_2_OH)_2_} and sulfate ion (SO_4_).

**Fig 3:**
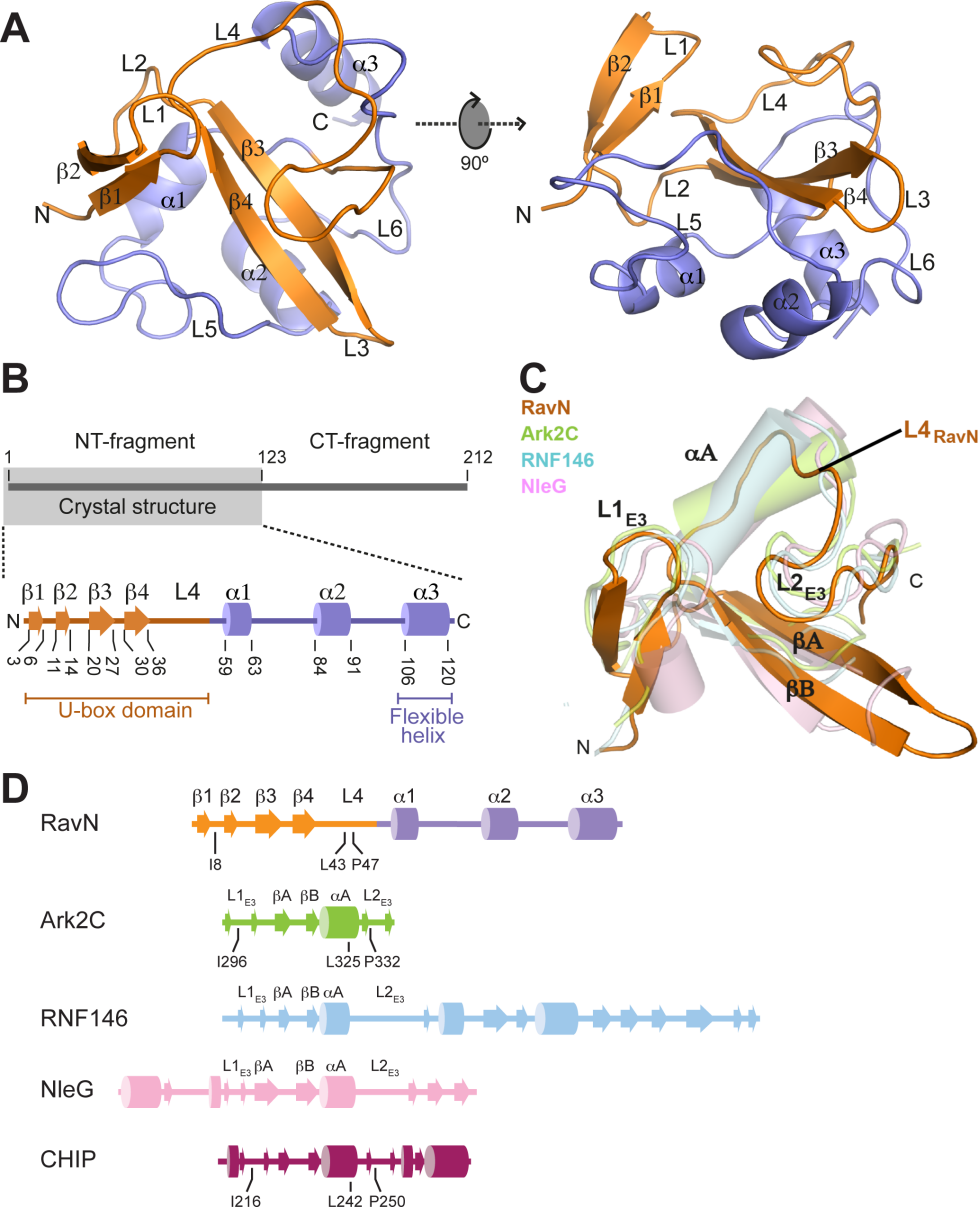
The E3 ligase domain of RavN has a contorted U-box fold. (A) Schematic representation of the structure of RavN_1-123_ as ribbon diagram displayed in two orientations (rotated by 90° along the *x* axis). Secondary elements are indicated as spirals (helices) or arrows (beta strands), with the RING/U-box motif colored in orange and the C-terminal structure colored in slate. (B) Topology diagram of RavN_1-123_ with the same color scheme as shown in (A). Numbers indicate amino acid residues. (C) Superimposition of the U-box domain of RavN (RavN_1-55_, orange) with the RING domains of Ark2C (PDB 5D0I, light green) and RNF146 (PDB 4QPL, light blue), and the U-box domain of NleG (PDB 2KKX, light pink). Conserved structural elements shared among RING/U-box domains are labeled as L1_E3_, L2_E3_, αA, βA, and βB. The position of αA is occupied by loop L4 in RavN (L4_RavN_). (D) Topology diagrams of the structural homologs of RavN. The same color scheme is used as in (C) and in Fig 4A. The residues located at the E2 binding interface in RavN, Ark2C, and CHIP (as seen in Fig 4A) are indicated.

Analysis of the structure of RavN_1-123_ revealed the presence of a U-box-like fold spanning residues 1 to 55 (colored in orange in Fig 3A and 3B) followed by a domain of three alpha helices (α1-α3; colored in slate in Fig 3A and 3B). The U-box domain resembled the zinc-finger-containing structure present in all RING domains, but lacked the Zn^2+^-coordinating sites which are substituted by a network of stabilizing hydrogen bonds and salt bridges [11]. A search for structural homologs using the Dali server [45] confirmed that the closest match to this region of RavN were RING-finger/U-box domains, including that of the human Ark2C RING E3 ligase (PDB 5D0I, with Z-score 3.1 and RMSD 2.3 Å over 44 residues), the human RNF146 RING E3 ligase (PDB 4QPL, with Z-score 2.7 and RMSD 2.4 Å over 41 residues), and the *E*. *coli* NleG U-box E3 ligase (PDB 2KKX, with Z-score 2.0 and RMSD 2.6 Å over 46 residues) (Fig 3C and 3D) [46–48].

The canonical core of RING-finger/U-box domains is composed of a central β-hairpin (βA and βB) followed by one α-helix (αA) and flanked by two surface-exposed loops (L1_E3_ and L2_E3_) in a cross-brace arrangement [11] (Fig 3C and 3D). However, in the case of the RavN U-box domain, the position corresponding to αA is occupied by a segment of the loop L4 (residues 37–42; L4_RavN_) that connects the central β-hairpin with the equivalent loop L2_E3_ without altering its relative orientation (Fig 3C and 3D). In fact, for all active RING-finger/U-box domains, the L1_E3_, L2_E3_ and αA elements create a conserved hydrophobic surface that serves as the docking site for E2 binding, as investigated for RavN below. The remaining three alpha helices (α1, α2 and α3) and the connecting loops (L5 and L6) are folded as a “cup” or “goblet” (colored slate in Fig 3A) that surrounds the base of the U-box domain. Nonetheless, comparison between the three RavN_1-123_ molecules found in the asymmetric unit shows a significant conformational flexibility in helix α3 (see below).

### The mode of E2 binding by RavN has been preserved throughout evolution

**Fig 4:**
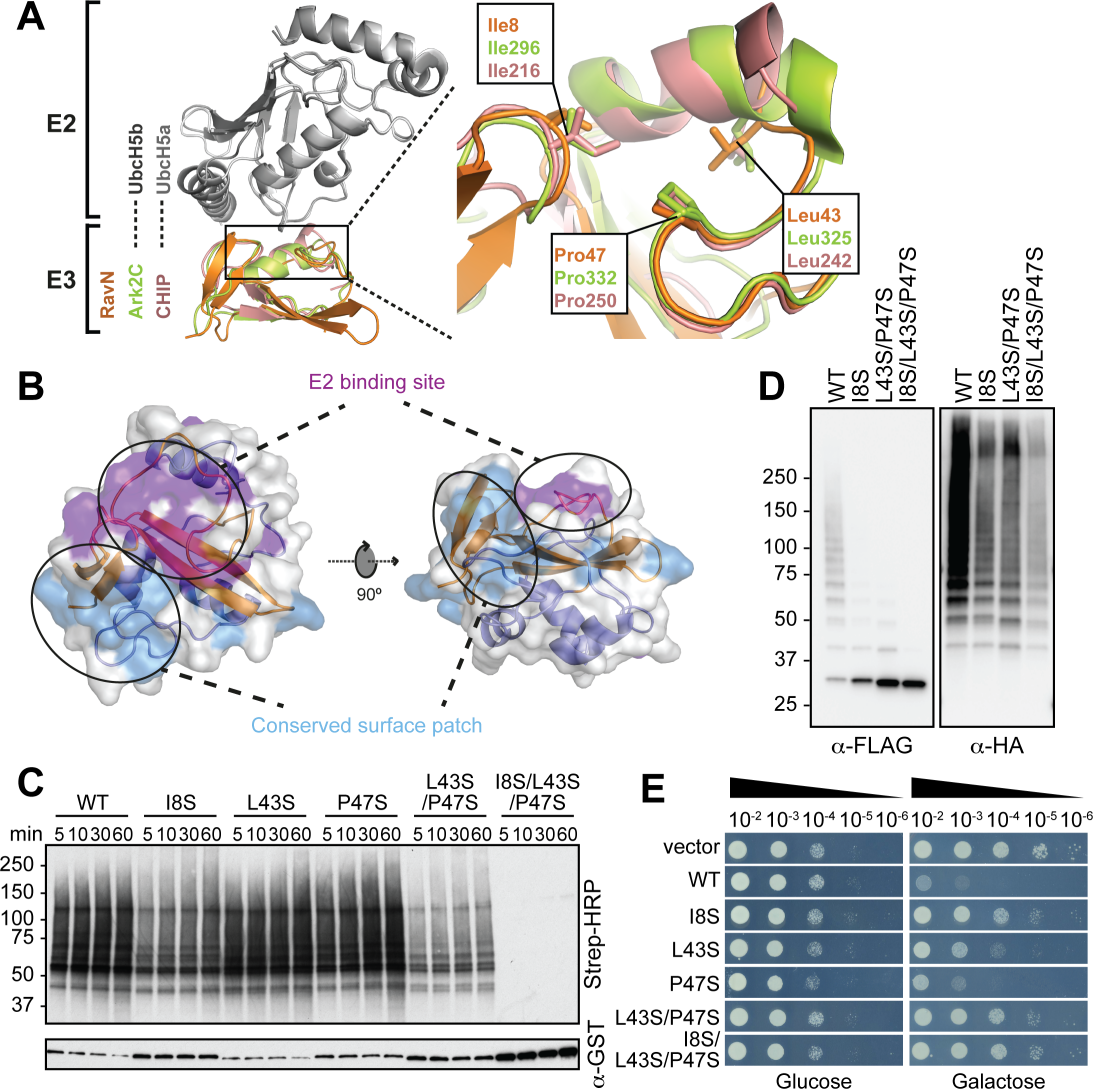
The U-box-E2 binding interface is conserved in RavN. (A) Superimposition of RavN_1-55_ (orange) and two related E3s (RING domain of Ark2C, light green; PDB 5D0K; U-box domain of CHIP, dark pink, PDB 2OXQ) in complex with their respective E2s (UbcH5b and UbcH5a) shown in gray. A close-up view highlights residues in RavN that are homologous to those located at the core of the E2 binding site in the other two E3s. (B) Residues conserved in all ten RavN homologs (labeled with an asterisk in S2A Fig) cluster into two patches at the surface of the RavN_1-123_ molecule (indicated by ovals), one of which overlaps with the proposed E2 binding interface. (C) The effect of interface residue substitutions in RavN was determined in the *in vitro* reconstitution assay using UbcH5a as E2. The formation of poly-ubiquitylated RavN was detected after 5, 10, 30, and 60 minutes with HRP-conjugated streptavidin. The total amount of GST-RavN in each reaction was determined by immunoblot using anti-GST antibody. WT: wild-type RavN. (D) Cell-based ubiquitylation assay. FLAG-tagged RavN variants simultaneously produced with HA-tagged ubiquitin in HEK293T cells were immuno-precipitated, and poly-ubiquitylation was detected by immunoblot using anti-HA antibody (right). Total amount of FLAG-tagged RavN or RavN variants was detected by immunoblot using anti-FLAG antibody (left). (E) Inhibition of yeast growth is dependent on the E3 ligase activity of RavN. *S*. *cerevisiae* INVSc1 cells containing plasmids encoding wild-type (WT) RavN or the indicated RavN variants were grown in the presence of either glucose (to repress *ravN* expression) or galactose (induces *ravN* expression), and cell growth was determined after 48 hours by spotting serial dilutions.

To determine the mode of E2 binding within RavN, we compared its structure with that of Ark2C and another U-box-containing protein, CHIP, which have recently been crystallized in complex with the E2 enzymes UbcH5b and the zebrafish ortholog of UbcH5a (Ark2C, PDB 5D0K; CHIP, PDB 2OXQ) (Fig 4A) [47, 49]. Superimposition of their structures with that of RavN revealed the presence of three residues in RavN (Ile8, Leu43 and Pro47) that create a surface area analogous to the E2 binding regions present in Ark2C and CHIP (Fig 4A and S4 Fig), suggesting that these residues could be important for E2 binding and, thus, E3 ligase activity of RavN. Notably, Ile8, Leu43, and Pro47 are preserved among all known RavN homologs from different *Legionella* species (S2A Fig). Closer examination revealed that most of the residues that are conserved in all RavN homologs (marked with asterisk in S2A Fig) are clustered in two neighboring patches on the surface of RavN_1-123_, with one patch overlapping with the putative E2 binding interface (Fig 4B).

We generated RavN variants (RavN^I8S^, RavN^L43S^, and RavN^P47S^) in which each interface residue was individually replaced by serine, as well as a double mutant (RavN^L43S/P47S^) and a triple mutant (RavN^I8S/L43S/P47S^), and examined their E3 ligase activity in the *in vitro* reconstitution assay (Fig 4C). As described above, the presence of wild-type RavN resulted in robust poly-ubiquitylation as soon as five minutes into the reaction, and the level of ubiquitylation approached saturation after one hour of incubation. Of the three variants with single residue substitution, RavN^I8S^ displayed the most notable decrease in the E3 ligase activity. RavN^L43S/P47S^ showed an even more dramatic decrease in poly-ubiquitylation, and the E3 ligase activity of RavN^I8S/L43S/P47S^ was almost completely abolished, with little to no poly-ubiquitylation detectable even after one hour of incubation (Fig 4C). Importantly, using circular dichroism (CD) spectroscopy, we found no evidence for a change in the secondary structure content of RavN^I8S/L43S/P47S^ compared to wild-type RavN (S5A and S5B Fig), making it unlikely that the decrease in E3 ligase activity was the result of a failure to fold properly.

To evaluate the E3 ligase activity of RavN and its variants upon exposure to mammalian cell content, FLAG-tagged versions of the proteins were simultaneously produced with hemagglutinin (HA)-tagged ubiquitin in transiently transfected HEK293T cells, and ubiquitylated species were detected using anti-HA antibody after anti-FLAG immuno-precipitation (Fig 4D). While FLAG-RavN showed robust levels of poly-ubiquitylation (similar to Fig 1C), the substitution of either one (FLAG-RavN^I8S^) or two residues (FLAG-RavN^L43S/P47S^) within the putative E2 binding site was sufficient to severely reduce the poly-ubiquitylation activity of the proteins. Substituting all three residues nearly abolished the poly-ubiquitylation levels of FLAG-RavN^I8S/L43S/P47S^ (Fig 4D and S5C Fig). This result indicated that RavN ubiquitylation was dependent on complex formation with an E2 enzyme. The attenuation of ubiquitylation of FLAG-RavN^I8S/L43S/P47S^ also provided further evidence that the initial observation of RavN ubiquitylation upon incubation with cell lysate (Fig 1A) was primarily a consequence of its auto-ubiquitylation rather than the activity of host cell E3 ligases.

Overproduction of RavN did not cause any detectable cytotoxicity in mammalian cells (S1D Fig), but strongly attenuated growth of budding yeast on media plates (Fig 4E). Similar observations have been made for many other microbial effector proteins that interfere with signaling pathways that are highly conserved within eukaryotes [50]. We thus explored whether the attenuation of yeast growth in the presence of RavN was a result of its E3 ligase activity. Whereas production of wild-type RavN severely reduced growth of *S*. *cerevisiae* INVSc1 on media plates containing galactose, substitution of individual interface residues (RavN^I8S^, RavN^L43S^, and RavN^P47S^) of RavN resulted in a partial recovery of yeast growth. *S*. *cerevisiae* producing RavN variants with substitutions in either two (RavN^L43S/P47S^) or three (RavN^I8S/L43S/P47S^) interface residues grew at levels comparable to cells producing no RavN (vector control). Thus, inhibition of yeast growth upon RavN overproduction was most likely a result of its E3 ligase activity. Taken together, our cell-based and *in vitro* assays confirmed that even though the three-dimensional structure of RavN has been significantly altered during evolution compared to related U-box E3 ligases, the E2-E3 interface has remained conserved and plays a critical role for RavN’s function.

### The flexible α3 helix in RavN is dispensable for its E3 ligase activity

**Fig 5:**
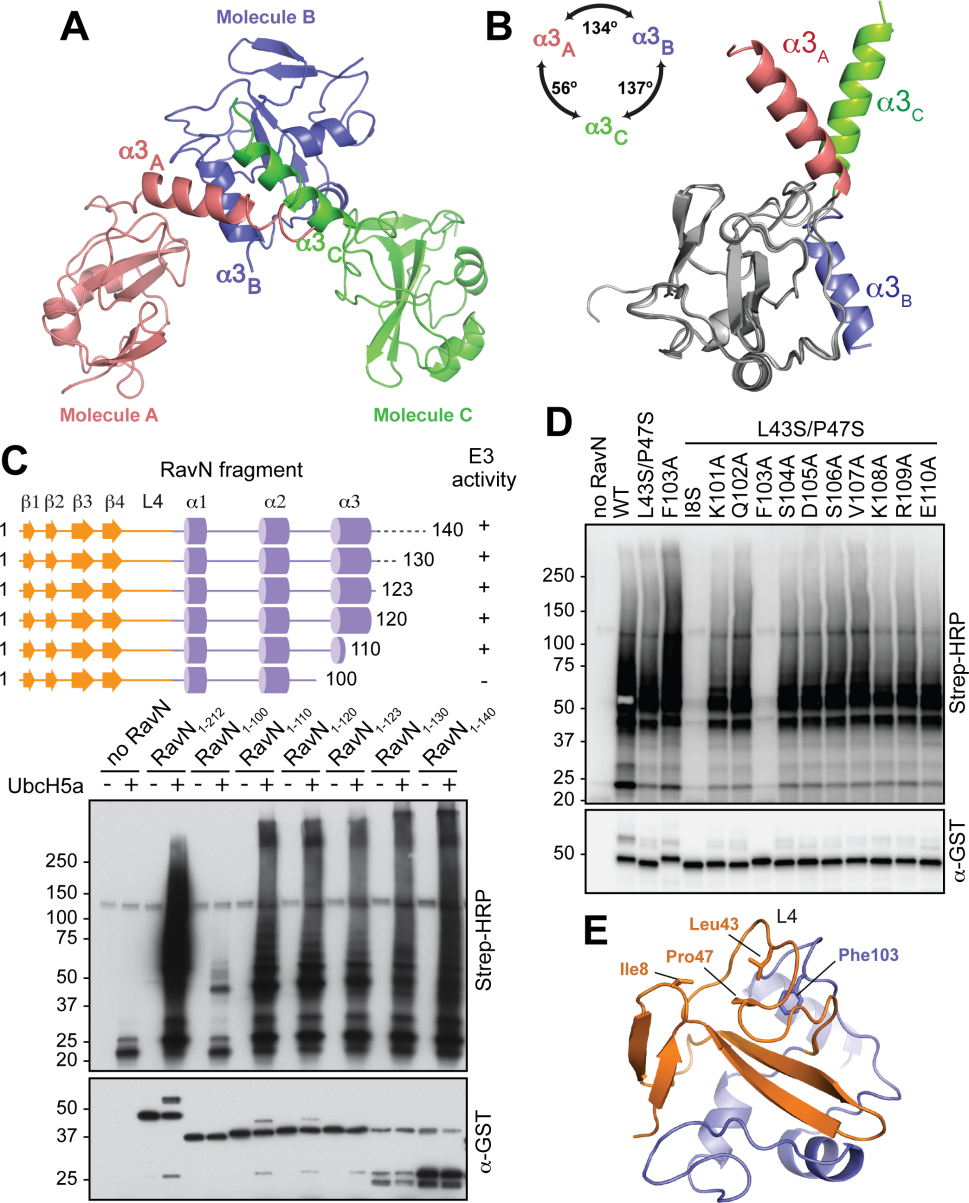
The flexible α3 helix is not required for the E3 ligase activity of RavN. (A) Each asymmetric unit within the protein crystal contained a RavN_1-123_ molecule (shown as red, slate and green ribbon diagrams) that differed with respect to the orientation of their helix α3 (α3_A_, α3_B_, and α3_C_). (B) Superimposition of the three conformations of RavN_1-123_ found in the asymmetric unit. Structures are presented as ribbon diagrams, with the overlaid part colored in gray and the variable α3 helix colored in red, slate and green. (C) Top: Schematic representation of RavN fragments examined in the *in vitro* reconstitution assay (bottom) using UbcH5a as E2. Poly-ubiquitylated RavN was detected with HRP-conjugated streptavidin, and the total amount of GST-RavN present in each reaction was confirmed by immunoblot using anti-GST antibody. (D) Phe103 is important for E3 ligase activity of RavN. An *in vitro* reconstitution assay was used to determine the effect of the indicated amino acid substitutions within region 101–110 on the E3 ligase activity of RavN^L43S/P47S^. UbcH5a was added as E2 enzyme. The formation of poly-ubiquitin species was detected using HRP-conjugated streptavidin. The total amount of GST-RavN in each lane was determined by immunoblot using anti-GST antibody. (E) The position of Phe103 relative to the three E2 binding interface residues Ile8, Leu43, and Pro47 in RavN is shown. Color scheme as in Fig 3A.

Our domain mapping revealed that RavN_1-140_, but not RavN_1-100_, functioned as E3 ligase (Fig 2), suggesting that residues 101–140, which include helix α3 (residues 106–120), were critical for activity. Notably, owing to crystal packing, helix α3 adopted multiple orientations among the three RavN_1-123_ molecules present within the asymmetric unit (Fig 5A and 5B). The angles between different orientations of helix α3 ranged from 56° for α3_A∠C_ to 134° for α3_A∠B_ and 137° for α3_B∠C_ (Fig 5B). This suggested a significant degree of structural flexibility for α3 most likely due to its loose association with the remainder of the E3 ligase domain. To validate if α3 was required for the E3 ligase activity, we sequentially truncated RavN_1-140_ from its C-terminal end and assessed the resulting RavN variants for their E3 ligase activity (Fig 5C). The only fragment tested here that was unable to catalyze poly-ubiquitylation was again RavN_1-100_, which lacked the entire α3 helix and parts of the preceding loop. All other RavN variants, including the crystallographic construct RavN_1-123_, showed E3 ligase activity similar to RavN_1-140_. In fact, even a fragment containing only five residues of α3 (RavN_1-110_) was fully active. Thus, the flexible α3 helix is unlikely to be directly involved in the E3 ligase activity of RavN.

We then turned our attention to examining the role of the residues within region 101–110 since their removal from RavN_1-110_ resulted in a drastic reduction in E3 ligase activity (Fig 5C). We replaced each of the ten residues individually with alanine, and examined via *in vitro* reconstitution assays if the substitution further aggravated the partial E3 ligase defect of the previously analyzed RavN^L43S/P47S^ double mutant (Fig 4C). While nine of the resulting RavN triple mutants showed no further reduction in ubiquitin ligase activity (Fig 5D and S5D Fig), substitution of Phe103 with alanine eliminated the E3 ligase activity of RavN^L43S/P47S/F103A^ (lane 8) to a degree similar to that of the I8S substitution in RavN^I8S/L43S/P47S^ (lane 5). When introduced into wild-type RavN, mutation F103A caused a slight yet noticeable decrease in E3 ligase activity of RavN^F103A^ (S5D Fig). Notably, the secondary structure content of RavN^L43S/P47S/F103A^ was nearly indistinguishable to that of wild-type RavN (S5A and S5B Fig), indicating that the loss of E3 ligase activity upon F103A substitution in RavN^L43S/P47S/F103A^ was not caused by the inability of the protein to fold correctly. Since Phe103 is not located within the putative E2 binding interface but, instead, positioned underneath loop L4 (Fig 5E), this residue most likely stabilizes Leu43 and Pro47 during their interaction with E2s. Consistent with its importance for the E3 ligase activity of RavN, Phe103 is the only residue within region 101–110 that is conserved among all known RavN homologs (S2A Fig).

### Discovery and validation of novel E3 ligases from *L*. *pneumophila*

**Fig 6:**
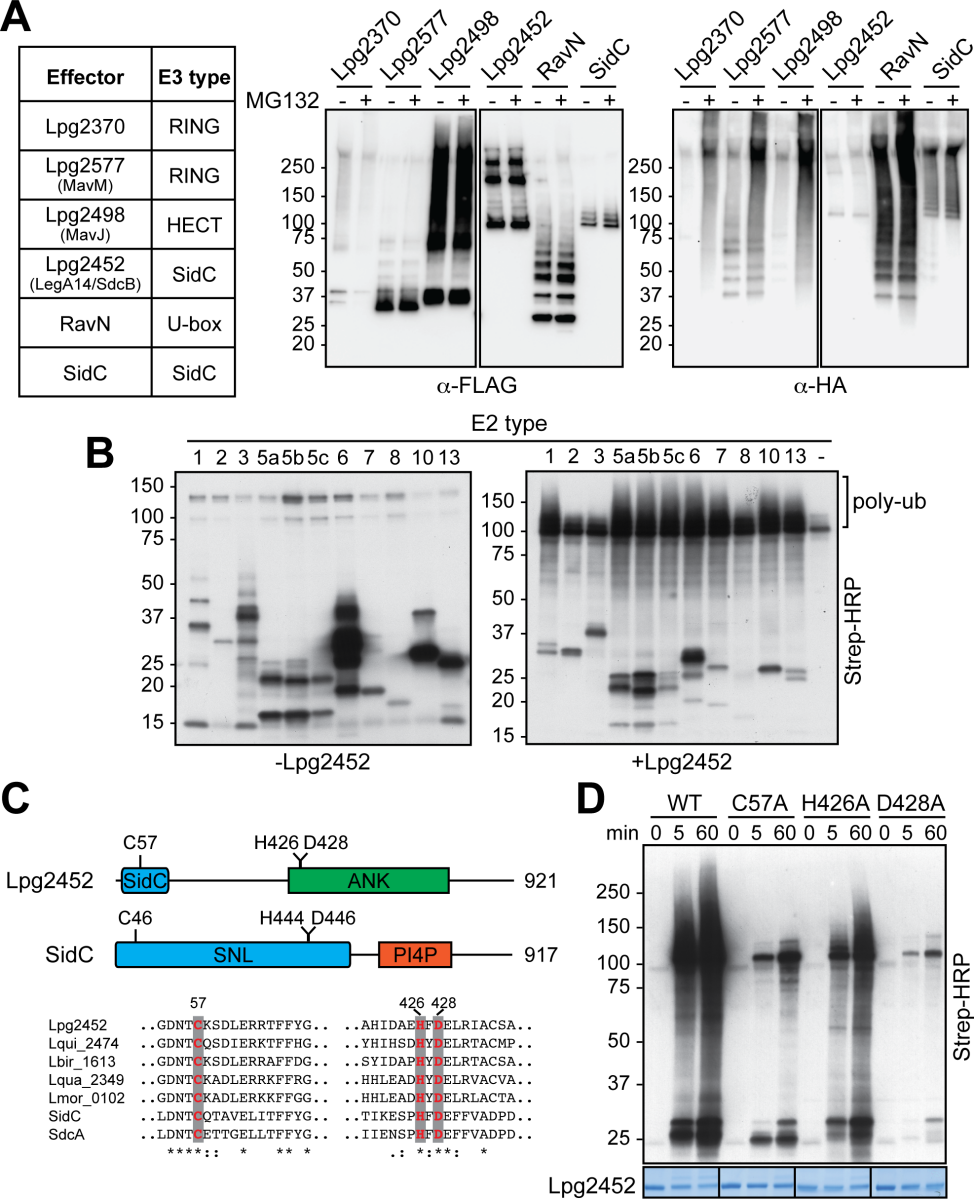
Identification and validation of previously unrecognized E3 ligase effectors. (A) Cell-based ubiquitylation assay. *L*. *pneumophila* effectors examined in the cell-based ubiquitylation assay are listed in the table with their predicted type of E3 indicated. FLAG-tagged effectors were co-produced with HA-tagged ubiquitin in transiently transfected HEK293T cells in the presence (+) or absence (-) of MG132 (proteasome inhibitor; 10 μM final concentration). The FLAG-tagged effectors were immuno-precipitated and detected by immunoblot using anti-FLAG antibody (left) while ubiquitylated species were visualized with an anti-HA antibody (right). (B) Lpg2452 catalyzes poly-ubiquitylation in *in vitro* reconstitution assays. The E2 enzymes present are listed on top of each lane. The lane labeled (-) lacks an E2. The blot on the left shows a control assay performed without Lpg2452. The position of poly-ubiquitylated products is indicated in bracket. (C) Top: Schematic representation of Lpg2452 and SidC. The region of Lpg2452 homologous to the E3 ligase domain of SidC is shown in blue; ANK, Ankyrin repeat region in green; SNL, SidC N-terminal E3 ligase in blue; PI4P, phosphatidylinositol 4-phosphate binding domain in orange. The location of the catalytic residues is indicated. Bottom: Primary sequence alignment of Lpg2452 with homologs from other *Legionella* species (Lqui_2474 from *L*. *quinlivanii*, Lbir_1613 from *L*. *birminghamensis*, Lqua_2349 from *L*. *quateirensis*, and Lmor_0102 from *L*. *moravica*; SidC and SdcA from *L*. *pneumophila* strain Philadelphia-1). The conserved catalytic triad residues (Cys57, His426, and Asp428 in Lpg2452) are highlighted. Identical residues are indicated with asterisks (*), highly conserved residues with colons (:), and weakly conserved residues with periods (.) (D) The Cys-His-Asp catalytic triad is critical for the E3 ligase activity of Lpg2452. *In vitro* reconstitution assay comparing the E3 ligase activity of wild-type (WT) Lpg2452 with that of the indicated catalytic mutants was performed using UbcH5a as E2. Poly-ubiquitylated species were detected at the indicated time points [in min] by HRP-conjugated streptavidin. The total amount of Lpg2452 and catalytic variants in each reaction was confirmed by Coomassie Blue staining (bottom).

The finding that RavN, despite lack of primary sequence similarity, showed remote structural homology and functional similarity to other U-box E3 ligases (Fig 3 and Fig 4) encouraged us to search for additional *L*. *pneumophila* effectors that may have taken a similar evolutionary route and that possess cryptic E3 ligase domains that may be identifiable based on secondary structure homology. Using pairwise comparison of profile hidden Markov models (HHpred [51]; see Material for details), we identified a total of four E3 ligase candidate effectors, all of which had previously been verified to be translocated substrates of the *L*. *pneumophila* T4SS [40, 41, 52, 53]. Lpg2370 and Lpg2577 (MavM) were predicted to possess homology to RING-type E3 ligases, whereas Lpg2498 (MavJ) was remotely similar to HECT-type E3 ligases (Fig 6A and S6 Fig). The National Center for Biotechnology Information (NCBI) database annotated Lpg2370, Lpg2577, and Lpg2498 as hypothetical proteins. Lpg2452 (LegA14) was annotated as an ankyrin repeat-containing protein in the NCBI database. Our *in silico* analysis revealed a striking secondary structure similarity of the N-terminal region of Lpg2452 with the *L*. *pneumophila* effectors SidC and SdcA (S6D Fig), raising the possibility that Lpg2452 is a previously unrecognized paralog of members from this unique E3 ligase family.

To examine if Lpg2452 or any of the other candidates functioned as bona fide E3 ligase, we analyzed them in the cell-based ubiquitylation assay (Fig 6A). HA-tagged ubiquitin was simultaneously produced with each of the FLAG-tagged effectors and, upon immuno-precipitation of the effectors, ubiquitylation was detected by SDS-PAGE and immunoblot analysis using anti-HA antibody (Fig 6A). Poly-ubiquitylated species were abundantly present in samples of all four FLAG-tagged E3 ligase candidates, with the poly-ubiquitylation signal of Lpg2370, Lpg2577, and Lpg2498, as well as RavN being strongly enhanced in the presence of MG132, a cell membrane-permeable inhibitor of the 26S proteasome that protects poly-ubiquitylated proteins, primarily proteins with Lys48-linked ubiquitylation, from degradation [54]. This suggested that the preferred products of these three *L*. *pneumophila* E3 candidates and RavN are likely Lys48-linked poly-ubiquitin chains. SidC was previously shown to catalyze Lys11- and Lys33-linked poly-ubiquitylation [35], explaining why the abundance of its poly-ubiquitylation was not further increased in the presence of MG132 (Fig 6A). A similar behavior was apparent for FLAG-tagged Lpg2452 upon MG132 treatment, suggesting that the type of poly-ubiquitylation catalyzed by Lpg2452 resembled that of its paralogs SidC and SdcA. Indeed, upon probing immuno-precipitated effectors with a Lys48-linkage-specific antibody, we detected extensive poly-ubiquitin chain formation for Lpg2370, Lpg2577, Lpg2498, and RavN, while no Lys48-linked poly-ubiquitin chains were generated by Lpg2452 and SidC (S7 Fig).

We subsequently focused our attention on Lpg2452 as it could be easily purified as a recombinant protein from *E*. *coli*. Upon addition to the *in vitro* reconstitution assay, Lpg2452 efficiently catalyzed the formation of poly-ubiquitylated products using a diverse repertoire of E2s (Fig 6B), including UbcH7, one of the preferred E2s of SidC [35]. Although the N-terminal domain of Lpg2452 showed only remote sequence homology to SidC and SdcA (S6D Fig), the Cys-His-Asp catalytic triad found in SidC and SdcA appeared to be mimicked by Cys57, His426, and Asp428 in Lpg2452 and its homologs from other *Legionella* species (Fig 6C). We generated Lpg2452 variants with alanine substitutions in each of these residues and examined their ability to catalyze poly-ubiquitylation *in vitro* using UbcH5a as the E2 enzyme. As shown in Fig 6D, wild-type Lpg2452 displayed strong poly-ubiquitin chain formation as soon as five minutes into the reaction, whereas the activity of each of the three catalytic variants was largely decreased, with the most severe reduction observed for Lpg2452^C57A^ and Lpg2452^D428A^. These results strongly suggest that Lpg2452 is in fact a previously unrecognized paralog of SidC and SdcA that utilizes a similar fold and catalytic mechanism to hijack the host ubiquitylation machinery.

## Discussion

In the present study, we discovered and subsequently characterized several previously unrecognized E3 ligases from *L*. *pneumophila*, including RavN and the SidC paralog Lpg2452 (or “SdcB”), thus expanding the E3 repertoire of this organism to ten. This unexpected abundance of a variety of structurally and functionally diverse ubiquitin ligases suggests that harnessing the host ubiquitylation machinery is of greater importance for the virulence program of *L*. *pneumophila* than was initially thought. Prior to our study, six *L*. *pneumophila* proteins had been experimentally confirmed to exploit host ubiquitylation. Four (LegU1, AnkB, LubX, and GobX) had been discovered based on primary sequence homology to known E3s [28, 30, 32], while the other two (SidC and the SidE family proteins) were identified based on structural data or functional assays [35, 36]. By also taking secondary structure similarity into consideration, we revealed several additional *L*. *pneumophila* effectors with E3 ligase activity that were missed in previous *in silico* studies due to the lack of primary sequence homology to known E3s.

Upon exposure to the cytosolic content of mammalian cells, RavN exhibited robust poly-ubiquitylation signals (Fig 1A through 1C), and we subsequently validated its E3 ligase activity in *in vitro* reconstitution assays (Fig 1D). This was an unexpected finding since RavN showed neither primary sequence nor secondary structure homology to known E3s. Yet, the crystal structure of RavN_1-123_ (Fig 3) revealed that its three-dimensional fold has residual resemblance to U-box E3 ligases, such as human Ark2C and *E*. *coli* NleG, providing evidence that RavN is indeed a bona fide E3. The similarity though was limited mainly to the central beta-sheet, suggesting that this effector has been acquired by *Legionella* through horizontal gene transfer early during evolution and then structurally altered over time to best fulfill its current function. Remarkably, despite this extensive structural adaptation, the residues in RavN (Ile8, Leu43, and Pro47) that comprise the interface for host E2 binding are conserved in all known *Legionella* RavN homologs and similar to those in other U-box E3s (Fig 4 and S2A Fig). Substitution of these residues significantly reduced the E3 ligase activity of RavN in a variety of assays (Fig 4), consistent with their importance for E2-ubiquitin binding. Phe103, another conserved residue in RavN homologs (S2A Fig) most likely provides structural support to the E2 binding interface (Fig 5E), and removal of this residue attenuated ubiquitylation activity of RavN (Fig 5D and S5D Fig) without altering its three-dimensional fold (S5A and S5B Fig). A phylogenetic tree built from the primary sequences of all known RavN homologs (S2B Fig) exactly paralleled that assembled from their encoding species’ entire genome sequences (S1A Fig), suggesting that *ravN* was most likely acquired by a common *Legionella* ancestor in a single horizontal gene transfer event.

The C-terminal domain (residues 124–212) of RavN, which was not part of the structure presented herein, is most likely involved in binding of a yet to be identified target protein in the cytosol (S1D and S1E Fig). Helix α3, which was dispensable for poly-ubiquitylation to occur (Fig 5C), displayed extensive conformational heterogeneity within the asymmetric unit of the protein crystal (Fig 5A and 5B). Our findings suggest a model where α3 may be an interconnecting linker that allows the adjacent N- and C-terminal domains of RavN to assume different orientations relative to each other (S8 Fig). The movement of the α3 helix could bring the target protein(s), presumably bound by the C-terminal domain, into close proximity with the E2-ubiquitin complex on the E3 domain, so that ubiquitin transfer can occur more effectively. A similar observation has been made for *Salmonella* SopA and *E*. *coli* NleL [17, 55], HECT domain-containing E3 ligases that undergo substantial conformational changes as part of their catalytic cycle.

Using secondary structure prediction models, we discovered four new *L*. *pneumophila* E3 ligase candidates (Lpg2370, Lpg2577, Lpg2498, and Lpg2452/SdcB) besides RavN that showed robust auto-ubiquitylation activity when produced in mammalian cells (Fig 6A). In fact, their levels of ubiquitylation were comparable to that of SidC, suggesting that they are active under the conditions tested here. Lpg2452/SdcB was further shown to catalyze poly-ubiquitylation in the *in vitro* reconstitution assay, and the Cys57-His426-Asp428 catalytic triad was critical for this activity (Fig 6D). This is a surprising finding given that the predicted fold homology between Lpg2452/SdcB and SidC-SdcA was limited to the N-terminal 120 amino acids (Fig 6C and S6D Fig). It did, however, further substantiate our hypothesis that even though *L*. *pneumophila* effectors may have undergone significant alteration at the primary and secondary structure level, features that are critical for their function have been preserved throughout evolution. Notably, although Lpg2452/SdcB and SidC-SdcA share a similar catalytic domain, their C-terminal regions are of entirely different composition. While SidC-SdcA possesses a membrane binding domain that recognizes phosphatidylinositol 4-phosphate (PI(4)P) [56], Lpg2452/SdcB contains an ankyrin repeat region that most likely mediates protein-protein interactions. Whether Lpg2452/SdcB and SidC-SdcA are functionally redundant during infection has yet to be determined, but their lack of synteny among sequenced *Legionella* strains favors that possibility (S9 Fig). Regardless of the identity of their host targets, the discovery of several new members of ubiquitin ligase families among *L*. *pneumophila* effectors underscores how important the ability to dynamically target the host ubiquitylation pathway is for *L*. *pneumophila* virulence.

## Materials and methods

### Strains and plasmids

*Legionella pneumophila* strain Lp02 and Lp03 (Lp02 *dotA3*) are thymidine-auxotroph derivatives of the isolate Philadelphia-1 [57]. Thymidine was supplemented at 100 μg/mL. All *L*. *pneumophila* strains are grown and maintained as described in [58].

Plasmids and oligonucleotides used in this study are listed in S1 and S2 Tables, respectively. The open reading frames (ORFs) encoding *L*. *pneumophila* effector proteins in plasmid pDONR221 were a kind gift of Ralph Isberg (Tufts University). pDONR221-*ravN*, *ravI*, *ankJ*, and *lpg2452* were transferred into the plasmid pDEST17 by *Gateway* Cloning (Life Technologies). pGEX-6p-1-*ravN* was generated by subcloning the ORF of *ravN* into pGEX-6p-1 (GE Healthcare) digested with *BamH*I and *Sal*I. C-terminal truncated *ravN* constructs were generated by inserting a stop codon at the corresponding nucleotide position in pGEX-6p-1-*ravN* using QuikChange site-directed mutagenesis (Agilent Technologies). pGEX-6p-1-*ravN*^*101-212*^ and pGEX-6p-1-*ravN*^*141-212*^ were generated by digesting pGEX-6p-1-*ravN*^*1-100*^ and pGEX-6p-1-*ravN*^*1-140*^ with *Bgl*II and *Sal*I, and ligated with pGEX-6p-1 digested with *BamH*I and *Sal*I.

pRK5-HA-Ubiquitin was obtained from Addgene (plasmid number: 17608). pcDNA5/FRO/TO-FLAG-*ravN* was generated by amplifying *ravN* with oligonucleotides [HindIII_FLAG_TEV_ravN_for_a, HindIII_FLAG_TEV_ravN_for_b, and XhoI_ravN_rev] that encode a single FLAG affinity tag, followed by restriction-mediated cloning via the *Hind*III and *Xho*I sites into the vector pcDNA^TM^5/FRO/TO (Invitrogen). pYES2-*ravN* was generated by subcloning *ravN* into the yeast expression vector pYES2/NTA (Invitrogen) digested with *BamH*I and *Xba*I. The pcDNA6.2-FLAG vector was generated by replacing the *emgfp* sequence in pcDNA6.2-EmGFP (Invitrogen) with the sequence encoding a single FLAG affinity tag using the *EcoR*V and *PspX*I site. Genes encoding *L*. *pneumophila* E3 ligase candidates were cloned into pcDNA6.2-FLAG via *Gateway* Cloning. Genes encoding RavN or effector proteins with single or multiple amino acid substitutions were generated by QuikChange site-directed mutagenesis (Agilent Technologies) using the respective primers (S2 Table). For protein crystallography experiments, the ORF encoding RavN and the N-terminal fragment of RavN (RavN_1-123_, residues 1–123) were cloned into the pGST-Parallel2 vector [59] with a cleavable N-terminal Glutathione S-transferase (GST) tag.

### Pulldown assays

The pDEST17 constructs were introduced into *E*. *coli* strain BL21(DE3), and the encoded His_6_-tagged effector proteins were produced in the presence of 0.2 mM IPTG for 16 hours at 20°C. Cells were harvested by centrifugation at 5,000 rcf, and pellets were suspended in lysis buffer [1X Phosphate-buffered saline (PBS), 1 mM β-mercaptoethanol, protease inhibitor cocktail]. After mechanical lysis of bacterial cell using a cell breaker (Microfluidics M-110P), His_6_-tagged effector proteins were enriched on TALON Metal Affinity Resin (Clontech), and non-specifically bound proteins were removed by washing with Wash buffer [1X PBS, 1 mM β-mercaptoethanol, 10 mM imidazole]. Human embryonic kidney (HEK293T) cells (ATCC) were suspended in Wash buffer containing protease inhibitor, and lysed with 40–50 strokes in a dounce homogenizer. The post-nuclear supernatant was collected by centrifugation at 15,000 rcf for 10 min at 4°C. The pulldown assay was performed by incubating the TALON affinity resin containing 50 μg effector protein overnight at 4°C on a rotator with post-nuclear supernatant of HEK293T cells containing 5 mg of total protein. The resin was washed five times with Wash buffer with 0.05% Tween-20 and mixed with 2X SDS sample buffer. Eluted proteins were separated by SDS-PAGE and detected by silver staining (Bio-Rad) or on an immunoblot using anti-ubiquitin antibody.

### *In vitro* ubiquitylation

His_6_- or GST-tagged RavN or His_6_-tagged Lpg2452 were overproduced in the presence of 0.2 mM IPTG for 16 hours at 20°C and purified with TALON Metal Affinity Resin (Clontech) or Glutathione Sepharose 4B (GE Healthcare) following the manufacturer’s protocol. Proteins were dialyzed against 1X PBS with 1 mM β-mercaptoethanol. *In vitro* ubiquitylation was performed using the Ubiquitinylation kit (Enzo Life Sciences). In brief, a 10 μL reaction mixture containing 1X ubiquitinylation buffer, 5 mM Mg-ATP, 1 mM DTT, 2.5 μM biotinylated ubiquitin, and 100 nM E1 was combined with 1 μM to 2.5 μM of the various E2s and 1 μM purified E3 (RavN or Lpg2452). The reaction was incubated at 37°C for one hour or the indicated time, mixed with an equal volume of 2X SDS sample buffer, and incubated at 94°C for 10 min. The proteins were separated by SDS-PAGE, transferred onto a nitrocellulose membrane, and ubiquitylated products were detected with HRP-conjugated streptavidin.

### Cell-based ubiquitylation

2x10^5^ HEK293T cells were transiently transfected with pcDNA5/FRO/TO-FLAG-*ravN* or pcDNA6.2-FLAG constructs encoding FLAG-tagged effector proteins with pRK5-HA-ubiquitin. After overnight incubation, the cells were treated with or without 10 μM MG132 for six hours and were harvested by scraping. Cells were lysed in lysis buffer [50 mM Tris-HCl, pH 7.4, 150 mM NaCl, 1 mM EDTA, and 1% Triton X-100] at 4°C for 30 min, and the FLAG-tagged effector proteins were enriched on ANTI-FLAG M2 affinity gel (Sigma) according to the manufacturer’s protocol. The resin was mixed with SDS sample buffer, proteins were separated by SDS-PAGE, transferred onto a nitrocellulose membrane, and probed with anti-ubiquitin (Abcam or Life Technologies), anti-ubiquitin Lys48 linkage-specific antibody (Abcam), anti-HA (Abcam), or anti-FLAG (Sigma) antibody.

### Protein production and purification for crystallography

*E*. *coli* BL21(DE3) containing the pGST-Parallel2 vectors [59] encoding either RavN or RavN_1-123_ (residues 1–123) were grown in Luria-Bertani (LB) broth at 37°C, and gene expression was induced at an OD_600_ of 0.8 by the addition of 0.5 mM isopropyl-β-D-thiogalactopyranoside (IPTG). Cells were harvested after 16 hours of growth at 20°C. All following purification steps were performed at 4°C. The concentration of all purified proteins was calculated using the theoretical extinction coefficient.

RavN and RavN_1-123_ were purified using the following protocol. The cell pellet was resuspended in PBS-BME [10 mM Na_2_HPO_4_, 1.8 mM KH_2_PO_4_, 137 mM NaCl, 2.7 mM KCl, pH 7.4, 10 mM β-mercaptoethanol (BME)] and lysed by high-pressure homogenization at 27 Kpsi (Constant System Ltd). The cell lysate was subjected to centrifugation at 50,000 x g for 45 min, and the supernatant was loaded on a gravity column with glutathione-sepharose beads (GE Healthcare). The beads were washed with PBS-BME and subsequently with buffer A (50 mM Tris-HCl pH 8.0, 150 mM NaCl and 10 mM BME). Proteins were eluted from the beads after overnight incubation with tobacco etch virus (TEV) protease (in buffer A) which cleaves off the N-terminal GST-tag. Proteins of interest were diluted to a final concentration of 50 mM NaCl and passed over a 5 mL HitrapQ column (GE Healthcare) equilibrated in 50 mM Tris-HCl pH 8.0, 50 mM NaCl and 10 mM BME, and eluted using a 50–1,000 mM NaCl gradient. Finally, the protein was purified on a Superdex 200 16/60 size-exclusion column (GE Healthcare) in 50 mM Tris-HCl pH 7.4, 150 mM NaCl and 10 mM BME. Peak fractions were pooled, concentrated with an Amicon Centrifugal filter (Millipore), flash-frozen in liquid nitrogen, and stored at -80°C.

### Limited proteolysis and mass spectrometry

Proteolysis-resistant fragments of RavN were identified by incubating full-length RavN with trypsin at a ratio 250:1 for 90 minutes at 37°C. The resulting products were isolated by gel filtration on a Superdex 200 16/60 column. Two main peaks were observed and analyzed by mass spectrometry. Molecular weight determination was performed by MALDI-TOF analysis (Autoflex III Smartbeam, Bruker Daltonics, Bremen, Germany) by using sinapinic acid for sample preparation, and spectra were acquired in linear mode. The proteolytically resistant fragments had a molecular mass of 12,830 Da and 10,896 Da, which corresponded to the residues 1 to ~109 of RavN (including five additional residues (Gly-Ala-Met-Gly-Ser) from the TEV cleavage site) and the C-terminal region spanning amino acids ~118 to 212, respectively.

### Crystallization and structure determination

Crystals of RavN_1-123_ were obtained by hanging-drop vapor diffusion method at 18°C with a reservoir solution containing 1.5 M Ammonium sulfate, 0.1 M NaCitrate pH 5.4 and 0.2 M K/Na Tartrate. Crystallization drops contained 1 μL protein sample at 70 mg/mL, mixed with 1 μL reservoir solution. The crystals were cryoprotected by brief soaking in mother liquor containing 20–25% (vol/vol) glycerol and flash cooled in liquid nitrogen. For structure solution, crystals were incubated with the cryoprotectant solution containing NaI 400 mM for 4 minutes. Diffraction data of native and NaI derivative crystals were collected at 100 K either at the Diamond Light Source (Didcot, UK) beamline I04 or at the ALBA beamline XALOC (Barcelona, Spain) [60].

Data sets were integrated and scaled using XDS [61]. The crystals belong to space group C222 with three molecules in the asymmetric unit. Experimental phases were determined using single isomorphous replacement with anomalous signal (SIRAS). Determination of positions of iodide sites, phase calculations and solvent flattening were done using the SHELX suite [62] through the graphical interface HKL2MAP [63]. The protein model was built using ARP/wARP [64] which built 98% of the model. The final structure was obtained through iterative cycles of manual building and refinement using Coot [65] and Phenix [66]. The anomalous difference map allowed for the localization of 32 iodine sites in the NaI derivative. The Ramachandran statistics calculated by Molprobity are: 98.2%/1.8%/0% (favored/allowed/outliers). The crystallographic information is summarized in Table 1. Graphics presented in this manuscript were generated using the program PyMOL (http://www.pymol.org/).

### Yeast growth assay

Genes encoding RavN or RavN variants were expressed from pYES2/NTA (Invitrogen), a vector that contains a galactose-inducible (GAL1) promoter. *S*. *cerevisiae* strain INVSc1 cells were transformed with the resulting constructs using the lithium acetate/PEG transformation method described in the manufacturer’s protocol (Life Technologies). Transformants were grown at 30°C to saturation in synthetic liquid media containing 2% (w/v) glucose without uracil (-Ura). Cultures were normalized to an OD_600_ = 1, and 10-fold serial dilutions were spotted on synthetic media plates (-Ura) containing glucose (repressing) or galactose (inducing), and the growth was monitored after 48 hours of incubation.

### In silico analysis

The amino acid sequences of 296 *L*. *pneumophila* effector proteins were analyzed on the HHpred server using default parameters (https://toolkit.tuebingen.mpg.de/#/tools/hhpred) [51]. Effectors with predicted homology to known E3 ubiquitin ligases were selected, and regions of homology were aligned to determine the extent of overlap with the known E3 ubiquitin ligase domains.

### Accession numbers

Coordinates and structure factors have been deposited in the Protein Data Bank (http://www.rcsb.org/pdb) under ID code 5MIY.

## Supporting information

S1 Fig *ravN* distribution in different *Legionella* species. (A) Phylogenetic tree of the genus *Legionella* adapted from [44] showing the distribution of *ravN* being limited to *L*. *pneumophila* as well as two subclades (green dots and bold red lines). (B) Growth-phase-dependent production of RavN in either Lp02 or Lp02Δ*ravN* by immunoblot using anti-RavN antibody (GenScript). LidA served as loading control [67]. (C) RavN is not essential for intracellular replication. Human U937 macrophages were challenged with *L*. *pneumophila* strains Lp02, Lp03 (*T4SS*^*-*^), and Lp02Δ*ravN* for 2 hours, and bacterial colony-forming units were determined in a plating assay after 2, 24, 48, and 72 hours. Results are an average from two independent experiments. (D) RavN shows cytosolic distribution pattern. GFP-tagged RavN was produced in transiently transfected COS-1 cells, and its localization was analyzed by fluorescence microscopy. GFP was used as control. (E) RavN is enriched in the cytosolic fraction in infected macrophages. U937 cells challenged with Lp02 at an MOI of 100 for two hours were subjected to membrane fractionation. Calnexin and GAPDH served as marker proteins for the membrane (M) or cytosolic (C) fraction, respectively. P: post-nuclear supernatant.
(TIF)

S2 Fig Sequence alignment of RavN homologs. (A) Sequence alignment of RavN homologs from ten different *Legionella* species was performed with Clustal Omega [68]. Secondary structure elements of RavN_1-123_, as revealed by its crystal structure (Fig 3), are indicated as arrows (beta strands) and cylinders (alpha helices), and residues Ile8, Leu43, Pro47, and Phe103 are highlighted in yellow. Identical residues are indicated with asterisks (*), highly conserved residues with colons (:) and weakly conserved residues with periods (.). (B) Phylogenetic tree of RavN homologs based on sequence alignment shown in (A).
(TIF)

S3 Fig Electron density maps and determination of the oligomerization state of RavN_1-123_ in solution. (A) SEC-MALS analysis of RavN_1-123_. The MALS-based molecular weight profile for the elution peak is shown (blue solid line), along with the predicted MW of the monomer (14.5 kDa, blue dashed line) as reference. (B) Final 2Fo−Fc electron density calculated after the final refinement run (contoured at the 2σ level) (blue), with the final RavN model overlaid. (C) The anomalous difference electron-density map contoured at 5.0 σ showing the signals of the Iodine scattering atoms.
(TIF)

S4 Fig The E2 binding interface of RavN and CHIP. The E2 binding interface of RavN and CHIP (circled) are similar with respect to their topology and electrostatic potential, with positively (blue) and negatively (red) charged residues highlighted. The displayed electrostatic potential was calculated using APBS [69].
(TIF)

S5 Fig RavN mutant analysis. (A) Circular dichroism spectra of wild-type RavN, RavN^I8S/L43S/P47S^, and RavN^L43S/P47S/F103A^. The spectra were plotted with Molar Ellipticity θ (in deg x cm^2^ x dmol^-1^) against wavelength (in nm). (B) *In vitro* ubiquitylation assay using untagged RavN, RavN^I8S/L43S/P47S^, and RavN^L43S/P47S/F103A^ shown in (A). UbcH5a was added as E2 enzyme. Poly-ubiquitylation was detected by HRP-conjugated streptavidin (left), and total amounts of RavN present in each reaction were detected using RavN-specific antibody (right). (C) Ubiquitylated species in FLAG-RavN in Fig 4D were detected by anti-ubiquitin antibody. (D) The same blot as in Fig 5D but with shorter exposure time.
(TIF)

S6 Fig Sequence alignment of putative *L*. *pneumophila* E3 ligases with their homologs. All alignments were based on HHpred results. The top row indicates the predicted secondary structure of the query effector protein, and the bottom row displays the secondary structure of the template protein from the Protein Data Bank (H stands for helices, E for β-strand, and C for the coils; upper case letters means higher probability and lower case letters are lower probability). (A) Alignment of Lpg2370 with the RING-type E3 ligase FANCL (PDB ID 4CCG). (B) Alignment of Lpg2577 with the Bre1 RING finger domain (PDB ID 4R7E). The cysteine residues that form the zinc finger are highlighted in yellow. (C) Alignment of Lpg2498 with the HECT-type E3 ligase E6AP (PDB ID 1C4Z). (D) Alignment of the N-terminal 120 amino acid residues of Lpg2452 with SidC (PDB ID 4OOJ). The cysteine residue that forms the catalytic Cys-His-Asp triad is highlighted in yellow.
(TIF)

S7 Fig Poly-ubiquitylation catalyzed by *L*. *pneumophila* effectors. The immunoblot shown in Fig 6A was re-probed with anti-ubiquitin antibody (A) or antibody specific for Lys48-linked poly-ubiquitin chains (B). Poly-ubiquitylation signal of RavN is saturated (red) in blots after longer times of exposure.
(TIF)

S8 Fig Proposed model for RavN-mediated target ubiquitylation. Shown here is a proposed model for E2-Ub-RavN interaction and the dynamic motion of the flexible α3 helix that connects the E3 ligase domain of RavN (gray) with the putative target-binding C-terminal domain (yellow).
(TIF)

S9 Fig The synteny of *lpg2452*, *sidC*, and *sdcA* in different *Legionella* species. Overview of the presence (+) or absence (-) of *lpg2452*, *sidC*, and *sdcA* in different *Legionella* genomes. Note the apparent lack of synteny, except in the genome of *L*. *pneumophila* where *lpg2452* and *sidC/sdcA* coexist.
(TIF)

S1 Table Plasmids used in this study. (PDF)

S2 Table Oligonucleotides used in this study. (PDF)

S1 Methods (PDF)
