# Supplementary figures and images for: RavN is a member of a previously unrecognized group of Legionella pneumophila E3 ubiquitin ligases
Source: PLoS Pathog. 2018 Feb 7;14(2):e1006897. doi: 10.1371/journal.ppat.1006897 (PMC5819833; doi:10.1371/journal.ppat.1006897)

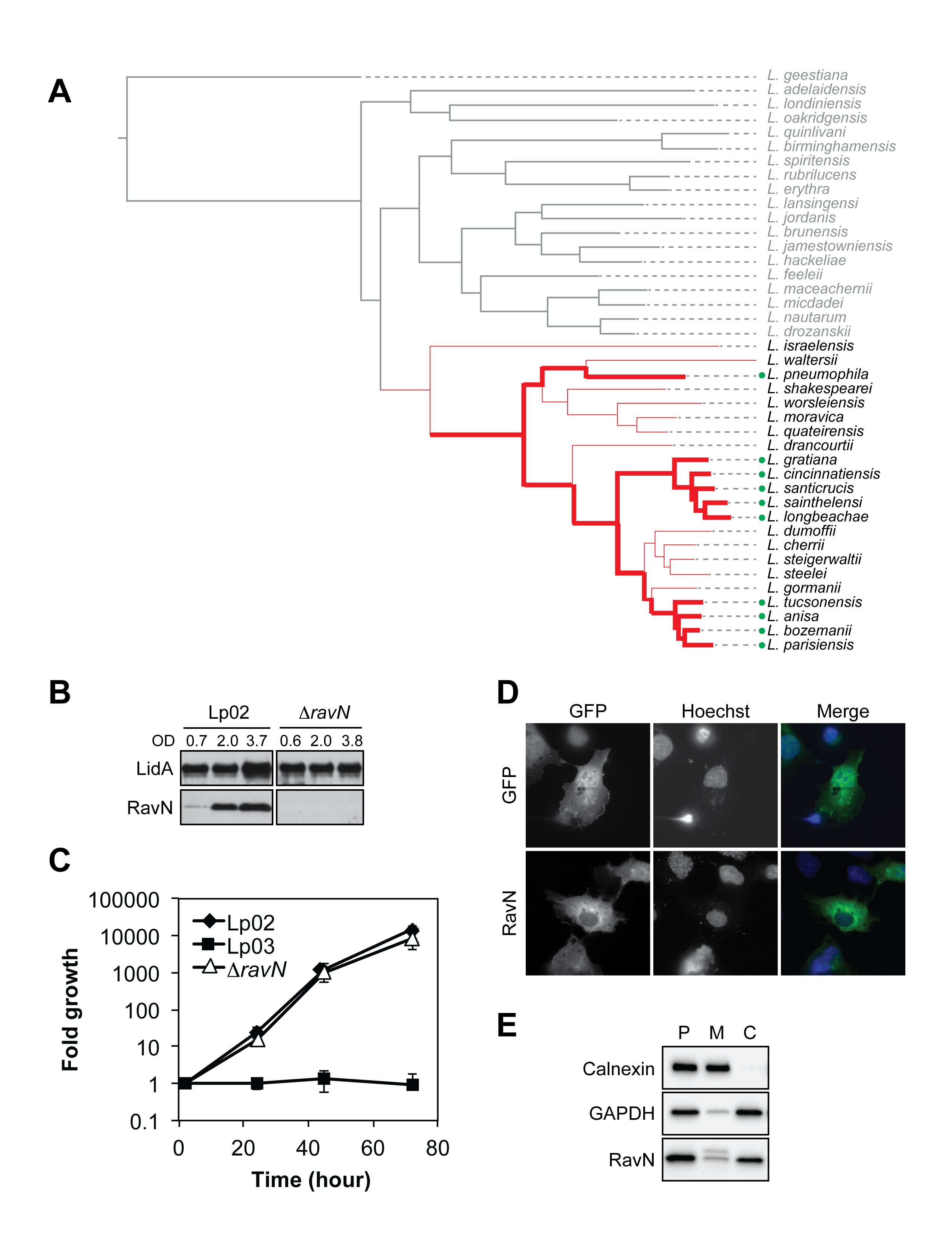

Supplement: S1 Fig — (A) Phylogenetic tree of the genus Legionella adapted from [44] showing the distribution of ravN being limited to L. pneumophila as well as two subclades (green dots and bold red lines). (B) Growth-phase-dependent production of RavN in either Lp02 or Lp02ΔravN by immunoblot using anti-RavN antibody (GenScript). LidA served as loading control [67]. (C) RavN is not essential for intracellular replication. Human U937 macrophages were challenged with L. pneumophila strains Lp02, Lp03 (T4SS-), and Lp02ΔravN for 2 hours, and bacterial colony-forming units were determined in a plating assay after 2, 24, 48, and 72 hours. Results are an average from two independent experiments. (D) RavN shows cytosolic distribution pattern. GFP-tagged RavN was produced in transiently transfected COS-1 cells, and its localization was analyzed by fluorescence microscopy. GFP was used as control. (E) RavN is enriched in the cytosolic fraction in infected macrophages. U937 cells challenged with Lp02 at an MOI of 100 for two hours were subjected to membrane fractionation. Calnexin and GAPDH served as marker proteins for the membrane (M) or cytosolic (C) fraction, respectively. P: post-nuclear supernatant. (TIF) [file ppat.1006897.s001.tif]

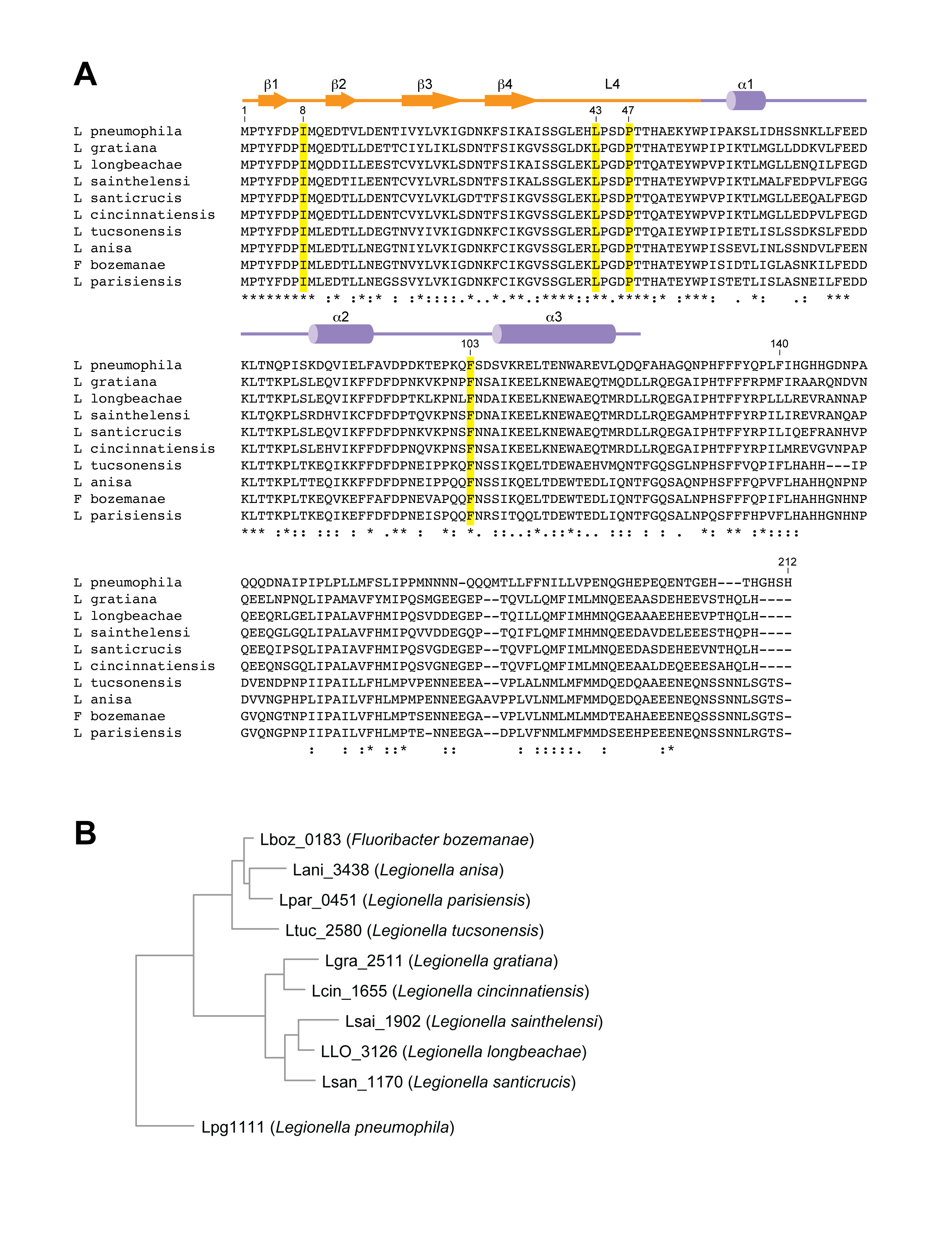

Supplement: S2 Fig — (A) Sequence alignment of RavN homologs from ten different Legionella species was performed with Clustal Omega [68]. Secondary structure elements of RavN1-123, as revealed by its crystal structure (Fig 3), are indicated as arrows (beta strands) and cylinders (alpha helices), and residues Ile8, Leu43, Pro47, and Phe103 are highlighted in yellow. Identical residues are indicated with asterisks (*), highly conserved residues with colons (:) and weakly conserved residues with periods (.). (B) Phylogenetic tree of RavN homologs based on sequence alignment shown in (A). (TIF) [file ppat.1006897.s002.tif]

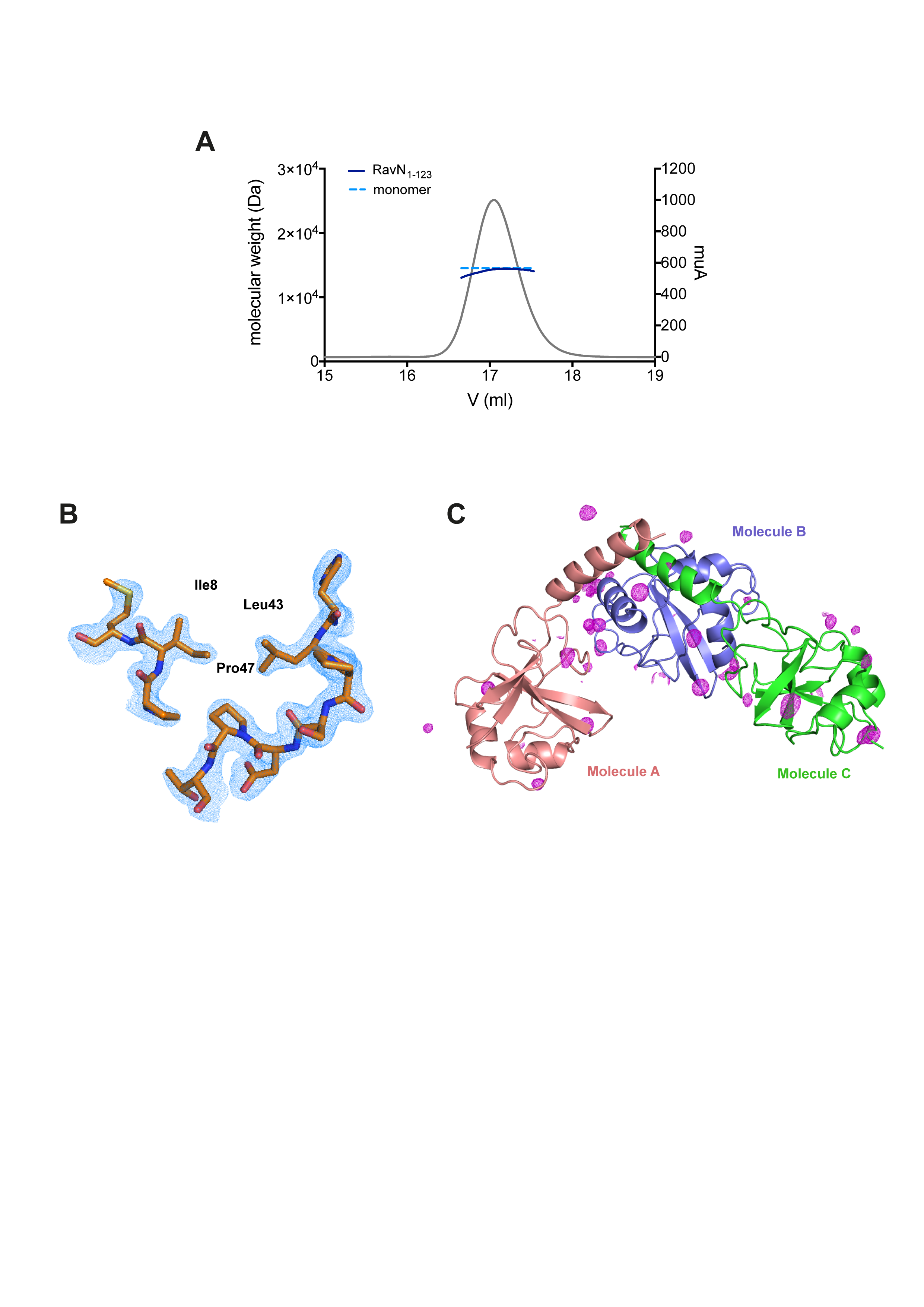

Supplement: S3 Fig — (A) SEC-MALS analysis of RavN1-123. The MALS-based molecular weight profile for the elution peak is shown (blue solid line), along with the predicted MW of the monomer (14.5 kDa, blue dashed line) as reference. (B) Final 2Fo−Fc electron density calculated after the final refinement run (contoured at the 2σ level) (blue), with the final RavN model overlaid. (C) The anomalous difference electron-density map contoured at 5.0 σ showing the signals of the Iodine scattering atoms. (TIF) [file ppat.1006897.s003.tif]

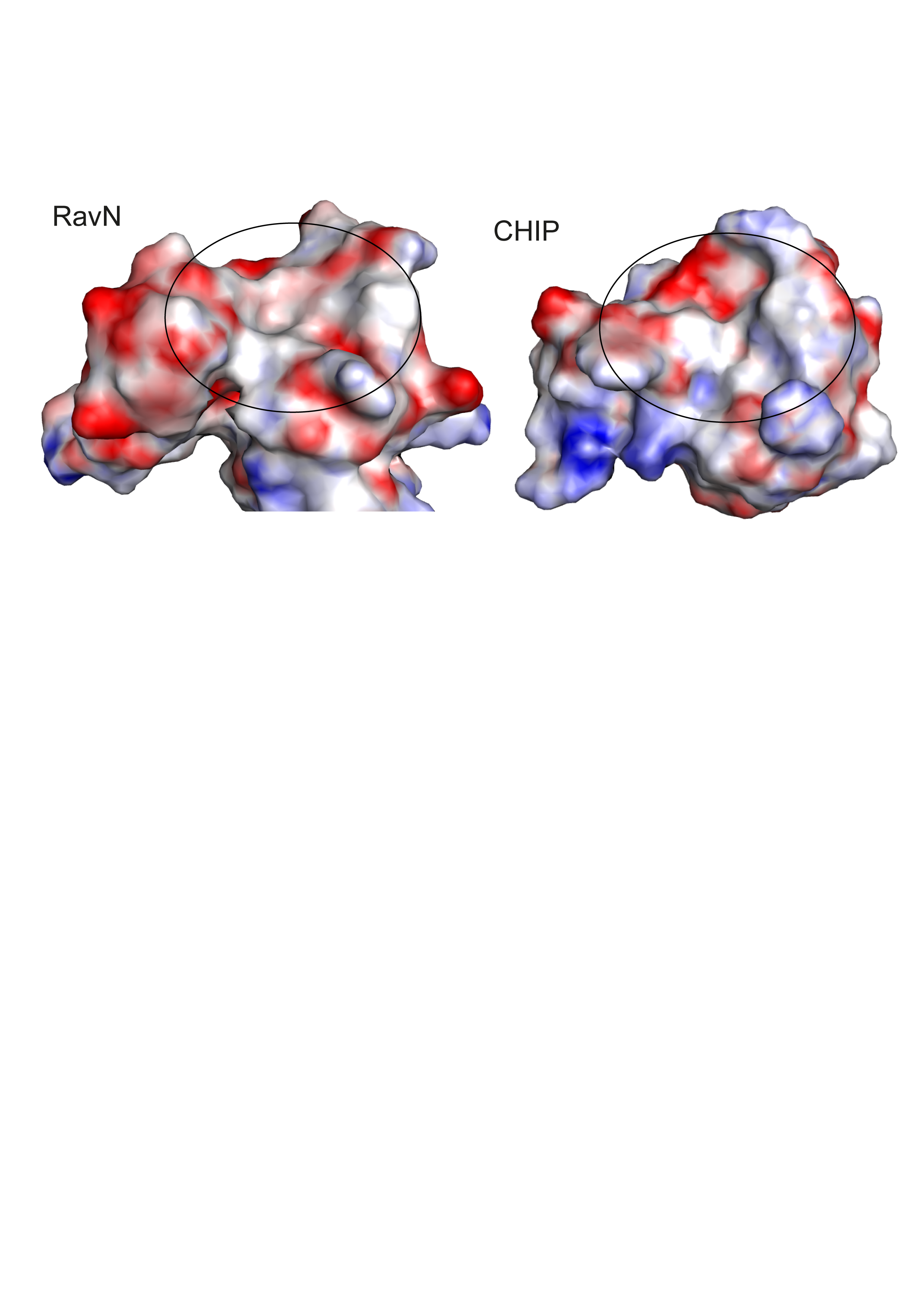

Supplement: S4 Fig — The E2 binding interface of RavN and CHIP (circled) are similar with respect to their topology and electrostatic potential, with positively (blue) and negatively (red) charged residues highlighted. The displayed electrostatic potential was calculated using APBS [69]. (TIF) [file ppat.1006897.s004.tif]

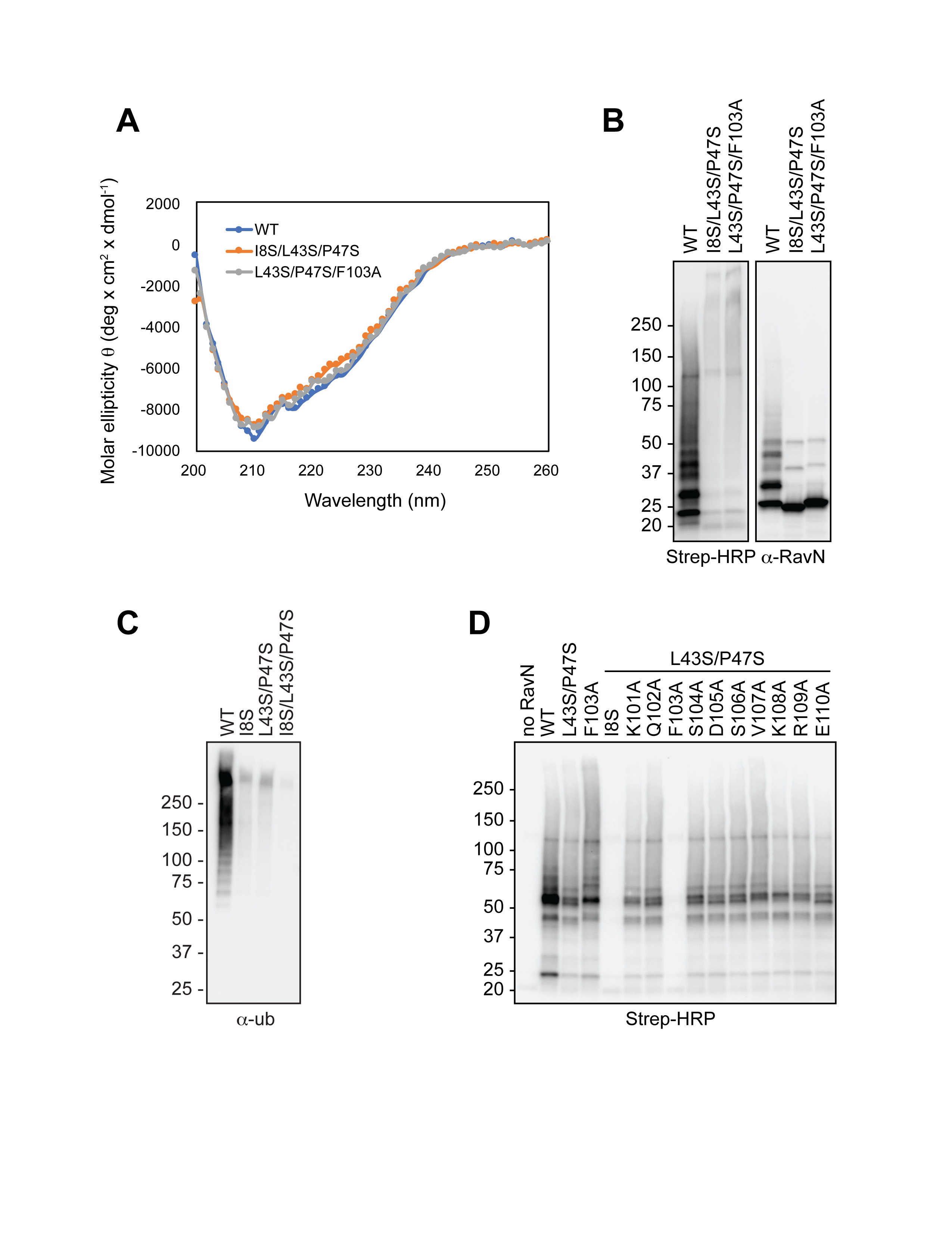

Supplement: S5 Fig — (A) Circular dichroism spectra of wild-type RavN, RavNI8S/L43S/P47S, and RavNL43S/P47S/F103A. The spectra were plotted with Molar Ellipticity θ (in deg x cm2 x dmol-1) against wavelength (in nm). (B) In vitro ubiquitylation assay using untagged RavN, RavNI8S/L43S/P47S, and RavNL43S/P47S/F103A shown in (A). UbcH5a was added as E2 enzyme. Poly-ubiquitylation was detected by HRP-conjugated streptavidin (left), and total amounts of RavN present in each reaction were detected using RavN-specific antibody (right). (C) Ubiquitylated species in FLAG-RavN in Fig 4D were detected by anti-ubiquitin antibody. (D) The same blot as in Fig 5D but with shorter exposure time. (TIF) [file ppat.1006897.s005.tif]

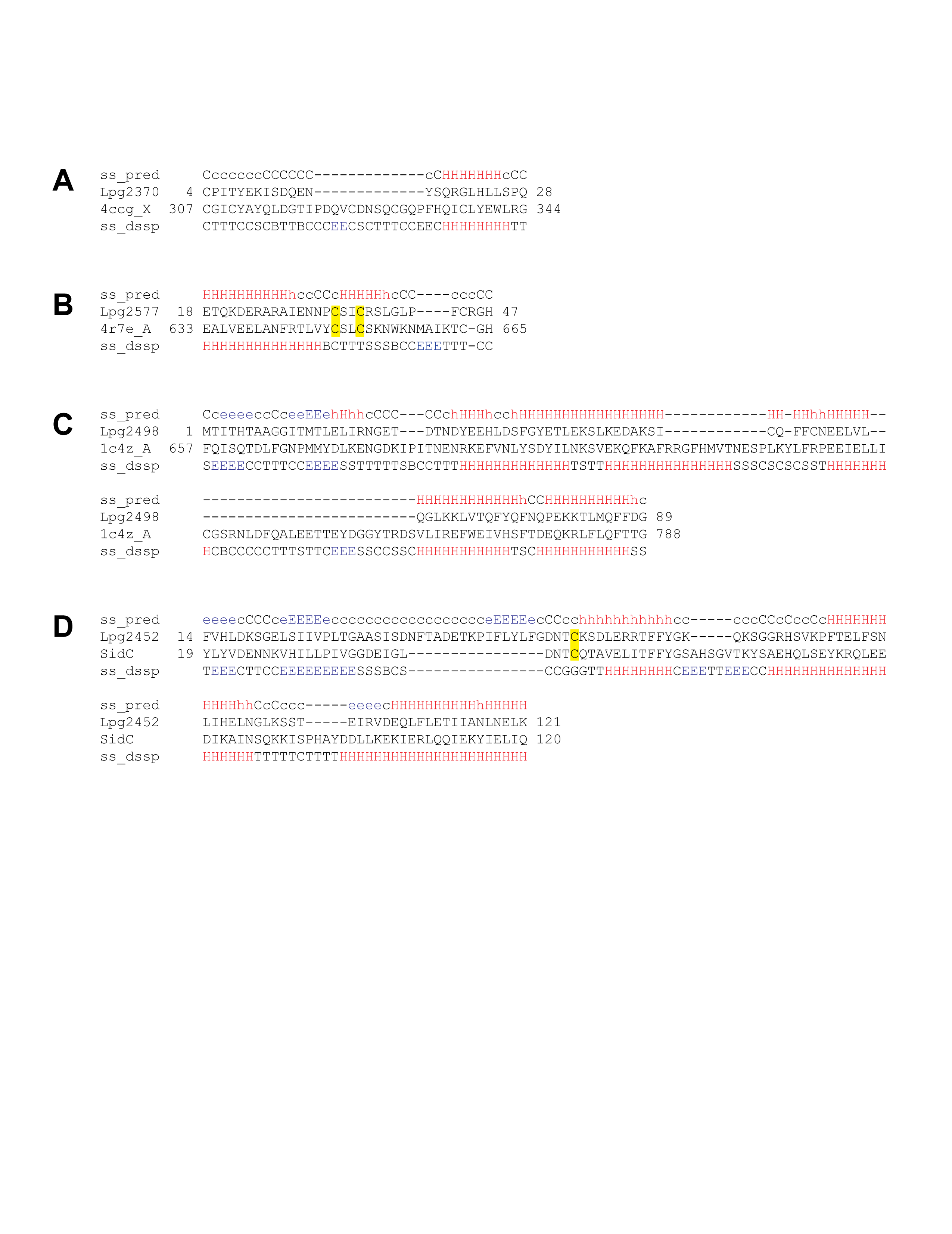

Supplement: S6 Fig — All alignments were based on HHpred results. The top row indicates the predicted secondary structure of the query effector protein, and the bottom row displays the secondary structure of the template protein from the Protein Data Bank (H stands for helices, E for β-strand, and C for the coils; upper case letters means higher probability and lower case letters are lower probability). (A) Alignment of Lpg2370 with the RING-type E3 ligase FANCL (PDB ID 4CCG). (B) Alignment of Lpg2577 with the Bre1 RING finger domain (PDB ID 4R7E). The cysteine residues that form the zinc finger are highlighted in yellow. (C) Alignment of Lpg2498 with the HECT-type E3 ligase E6AP (PDB ID 1C4Z). (D) Alignment of the N-terminal 120 amino acid residues of Lpg2452 with SidC (PDB ID 4OOJ). The cysteine residue that forms the catalytic Cys-His-Asp triad is highlighted in yellow. (TIF) [file ppat.1006897.s006.tif]

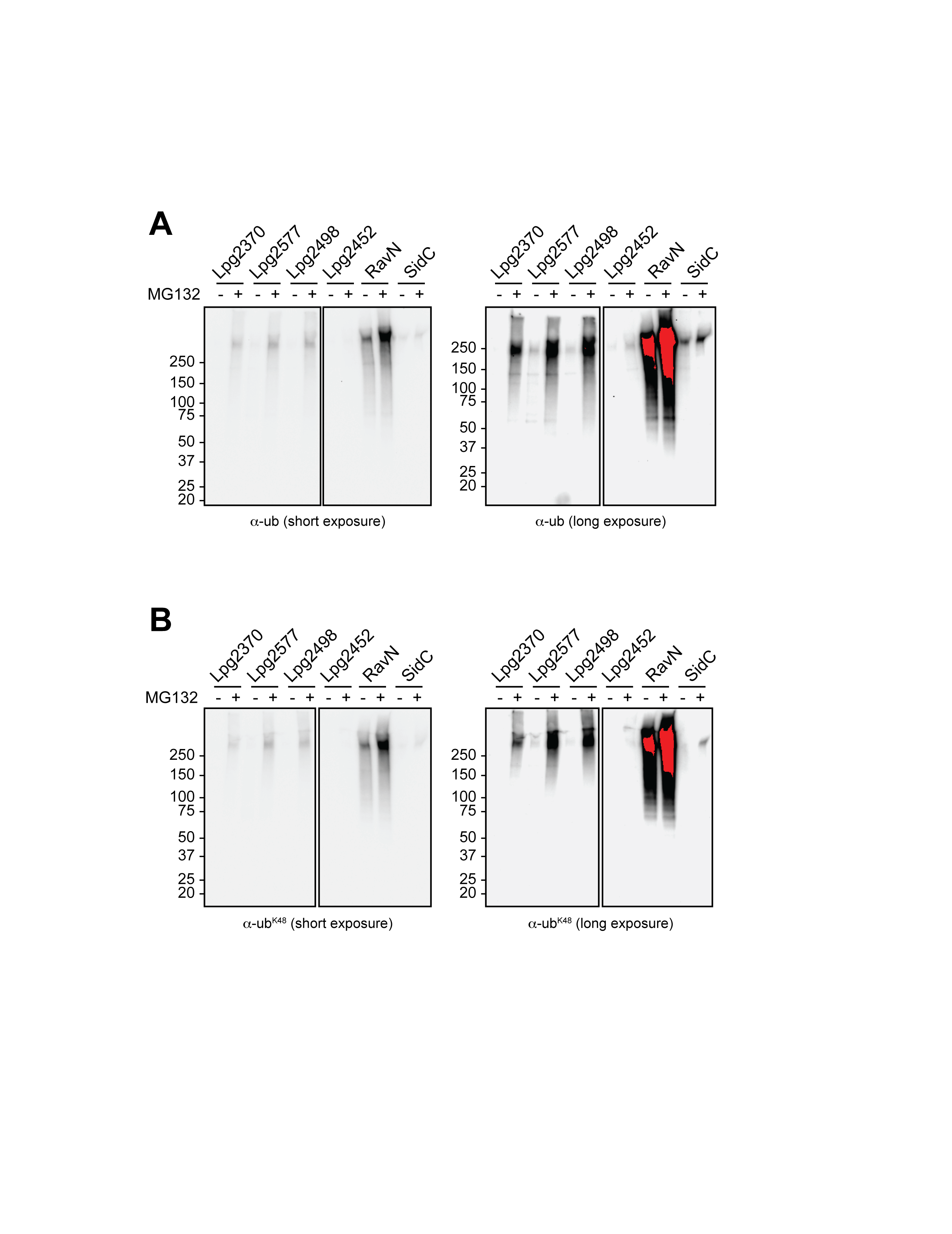

Supplement: S7 Fig — The immunoblot shown in Fig 6A was re-probed with anti-ubiquitin antibody (A) or antibody specific for Lys48-linked poly-ubiquitin chains (B). Poly-ubiquitylation signal of RavN is saturated (red) in blots after longer times of exposure. (TIF) [file ppat.1006897.s007.tif]

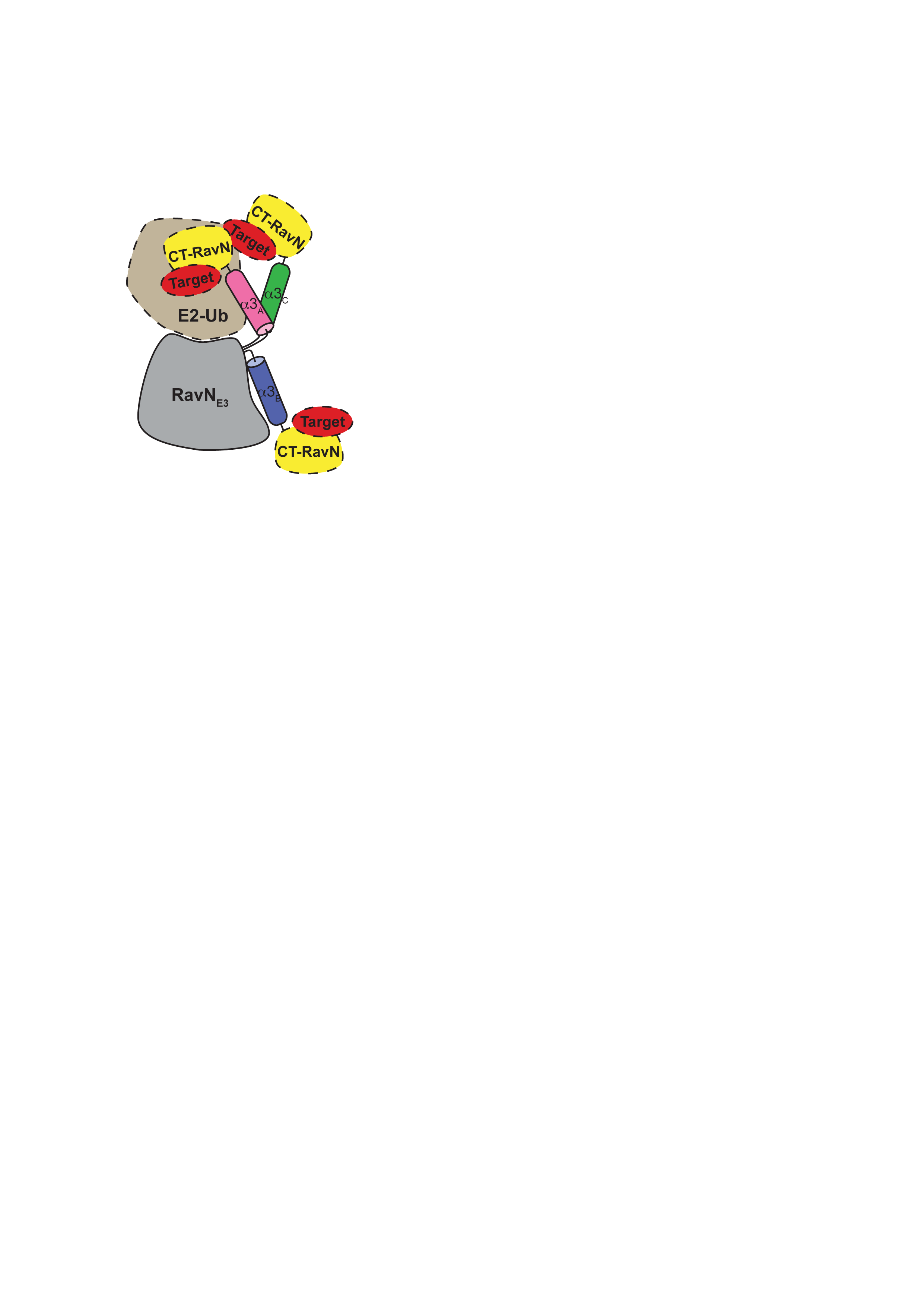

Supplement: S8 Fig — Shown here is a proposed model for E2-Ub-RavN interaction and the dynamic motion of the flexible α3 helix that connects the E3 ligase domain of RavN (gray) with the putative target-binding C-terminal domain (yellow). (TIF) [file ppat.1006897.s008.tif]

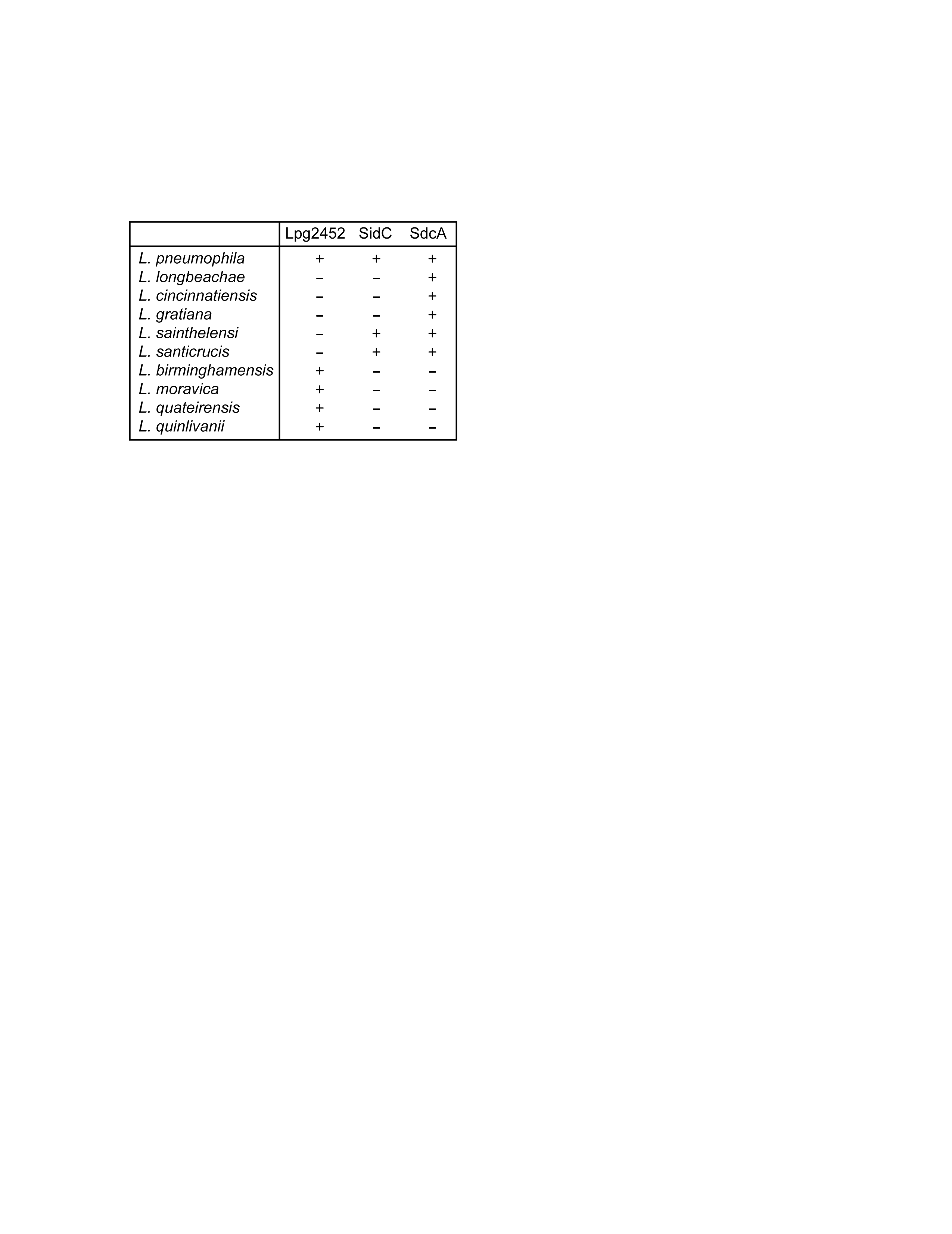

Supplement: S9 Fig — Overview of the presence (+) or absence (-) of lpg2452, sidC, and sdcA in different Legionella genomes. Note the apparent lack of synteny, except in the genome of L. pneumophila where lpg2452 and sidC/sdcA coexist. (TIF) [file ppat.1006897.s009.tif]
