# Supplementary material for: RavN is a member of a previously unrecognized group of Legionella pneumophila E3 ubiquitin ligases
Source: PLoS Pathog. 2018 Feb 7;14(2):e1006897. doi: 10.1371/journal.ppat.1006897 (PMC5819833; doi:10.1371/journal.ppat.1006897)
Supplement: S1 Table — (PDF) [file ppat.1006897.s010.pdf]

## Table S1. Plasmids used in this study.

| Name | Properties | Source |
|---|---|---|
| *E. coli* | | |
| pDEST17 | Gateway$^{TM}$ destination vector generating an N-terminal $His_6$ tag | Invitrogen |
| pDEST17-ravN | *ravN* in pDEST17 | This study |
| pDEST17-ravI | *ravI* in pDEST17 | This study |
| pDEST17-ankJ | *ankJ* in pDEST17 | This study |
| pDEST17-lpg2452 | *lpg2452* in pDEST17 | This study |
| pDEST17-lpg2452$^{C57A}$ | pDEST17-lpg2452 with mutation C57A | This study |
| pDEST17-lpg2452$^{H426A}$ | pDEST17-lpg2452 with mutation H426A | This study |
| pDEST17-lpg2452$^{D428A}$ | pDEST17-lpg2452 with mutation D428A | This study |
| pGEX-6p-1 | *E. coli* expression vector generating an N-terminal GST fusion | GE |
| pGEX-6p-1-ravN | *ravN* in pGEX-6p-1 | This study |
| pGEX-6p-1-ravN$_{1\text{-}100}$ | *ravN* residues 1-100 in pGEX-6p-1 | This study |
| pGEX-6p-1- ravN$_{1\text{-}140}$ | *ravN* residues 1-140 in pGEX-6p-1 | This study |
| pGEX-6p-1-ravN$_{101\text{-}212}$ | *ravN* residues 101-212 in pGEX-6p-1 | This study |
| pGEX-6p-1-ravN$_{141\text{-}212}$ | *ravN* residues 141-212 in pGEX-6p-1 | This study |
| pGEX-6p-1-ravN$_{1\text{-}110}$ | *ravN* residues 1-110 in pGEX-6p-1 | This study |
| pGEX-6p-1-ravN$_{1\text{-}120}$ | *ravN* residues 1-120 in pGEX-6p-1 | This study |
| pGEX-6p-1-ravN$_{1\text{-}123}$ | *ravN* residues 1-123 in pGEX-6p-1 | This study |
| pGEX-6p-1-ravN$_{1\text{-}130}$ | *ravN* residues 1-130 in pGEX-6p-1 | This study |
| pGEX-6p-1-ravN$^{I8S}$ | pGEX-6p-1-ravN with mutation I8S | This study |
| pGEX-6p-1-ravN$^{L43S}$ | pGEX-6p-1-ravN with mutation L43S | This study |
| pGEX-6p-1-ravN$^{P47S}$ | pGEX-6p-1-ravN with mutation P47S | This study |
| pGEX-6p-1-ravN$^{L43S/P47S}$ | pGEX-6p-1-ravN with mutations L43S, P47S | This study |
| pGEX-6p-1-ravN$^{I8S/L43S/P47S}$ | pGEX-6p-1-ravN with mutations I8S, L43S, P47S | This study |
| pGEX-6p-1-ravN$^{L43S/P47S/K101A}$ | pGEX-6p-1-ravN with mutations L43S, P47S, K101A | This study |
| pGEX-6p-1-ravN$^{L43S/P47S/Q102A}$ | pGEX-6p-1-ravN with mutations L43S, P47S, Q102A | This study |
| pGEX-6p-1-ravN$^{L43S/P47S/F103A}$ | pGEX-6p-1-ravN with mutations L43S, P47S, F103A | This study |
| pGEX-6p-1-ravN$^{L43S/P47S/S104A}$ | pGEX-6p-1-ravN with mutations L43S, P47S, S104A | This study |
| pGEX-6p-1-ravN$^{L43S/P47S/D105A}$ | pGEX-6p-1-ravN with mutations L43S, P47S, D105A | This study |
| pGEX-6p-1-ravN$^{L43S/P47S/S106A}$ | pGEX-6p-1-ravN with mutations L43S, P47S, S106A | This study |
| pGEX-6p-1-ravN$^{L43S/P47S/V107A}$ | pGEX-6p-1-ravN with mutations L43S, P47S, V107A | This study |
| pGEX-6p-1-ravN$^{L43S/P47S/K108A}$ | pGEX-6p-1-ravN with mutations L43S, P47S, K108A | This study |
| pGEX-6p-1-ravN$^{L43S/P47S/R109A}$ | pGEX-6p-1-ravN with mutations L43S, P47S, R109A | This study |
| pGEX-6p-1-ravN$^{L43S/P47S/E110A}$ | pGEX-6p-1-ravN with mutations L43S, P47S, E110A | This study |
| pGEX-6p-1-ravN$^{F103A}$ | pGEX-6p-1-ravN with mutation F103A | This study |
| pGST-Parallel2-ravN | *ravN* in pGST-Parallel2 | This study |
| pGST-Parallel2-ravN$_{1\text{-}123}$ | *ravN* residues 1-123 in pGST-Parallel2 | This study |
| *S. cerevisiae* | | |
| pYES2/NTA | *S. cerevisiae* expression vector containing the *URA3* gene and 2μ origin | Invitrogen |
| pYES2-ravN | *ravN* in pYES2/NTA | This study |
| pYES2-ravN$^{I8S}$ | pYES2-ravN with mutation I8S | This study |
| pYES2-ravN$^{L43S}$ | pYES2-ravN with mutation L43S | This study |
| pYES2-ravN$^{P47S}$ | pYES2-ravN with mutation P47S | This study |
| pYES2-ravN$^{L43S/P47S}$ | pYES2-ravN with mutations L43S, P47S | This study |
| pYES2-ravN$^{I8S/L43S/P47S}$ | pYES2-ravN with mutations I8S, L43S, P47S | This study |
| Mammalian cultured cells | | |
| pRK5-HA-Ubiquitin | Mammalian expression of HA tagged ubiquitin | Addgene |
| pcDNA5/FRO/TO | Inducible expression vector used in Flp-In$^{TM}$ T-Rex$^{TM}$ host cell lines | Invitrogen |
| pcDNA5/FRO/TO-FLAG-ravN | *ravN* with a N-terminal single FLAG tag in pcDNA5/FRO/TO | This study |
| pcDNA5/FRO/TO-FLAG-ravN$^{I8S}$ | pcDNA5/FRO/TO-FLAG-ravN with mutation I8S | This study |
| pcDNA5/FRO/TO-FLAG-ravN$^{L43S/P47S}$ | pcDNA5/FRO/TO-FLAG-ravN with mutations L43S, P47S | This study |
| pcDNA5/FRO/TO-FLAG-ravN$^{I8S/L43S/P47S}$ | pcDNA5/FRO/TO-FLAG-ravN with mutations I8S, L43S, P47S | This study |
| pcDNA6.2-FLAG | pcDNA6.2-EmGFP with *emgfp* sequence replaced by a single FLAG tag sequence | This study |
| pcDNA6.2-FLAG-lpg2370 | *lpg2370* in pcDNA6.2-FLAG | This study |
| pcDNA6.2-FLAG-lpg2452 | *lpg2452* in pcDNA6.2-FLAG | This study |
| pcDNA6.2-FLAG-lpg2498 | *lpg2498* in pcDNA6.2-FLAG | This study |

| | | |
|---|---|---|
| pcDNA6.2-FLAG-lpg2577 | *lpg2577* in pcDNA6.2-FLAG | This study |
| pcDNA6.2/N-EmGFP-DEST | Gateway destination vector generating an N-terminal EmGFP fusion | Invitrogen |
| pcDNA6.2/N-EmGFP-ravN | *ravN* in pcDNA6.2/N-EmGFP-DEST | This study |
| *Legionella* | | |
| pSR47S | R6K suicide vector ($Kan^r$ *sacB*) | [1] |
| pSR47S-ΔravN | pSR47S containing flanking regions of *ravN* | This study |

1. Merriam JJ, Mathur R, Maxfield-Boumil R, Isberg RR. Analysis of the Legionella pneumophila fliI gene: intracellular growth of a defined mutant defective for flagellum biosynthesis. Infection and Immunity. 1997;65(6):2497-501.
