# Supplementary material for: RavN is a member of a previously unrecognized group of Legionella pneumophila E3 ubiquitin ligases
Source: PLoS Pathog. 2018 Feb 7;14(2):e1006897. doi: 10.1371/journal.ppat.1006897 (PMC5819833; doi:10.1371/journal.ppat.1006897)
Supplement: S2 Table — (PDF) [file ppat.1006897.s011.pdf]

Table S2. Oligonucleotides used in this study.

**Name**	**Sequence** (5'→3', **bold**: nucleotide substitutions; underlined: restriction enzyme site; *italic*: *FLAG* tag)	**Purpose**
SalI_ravN_upst_for	AAAAGTCGACTTCAGCTGATAATTGGTTACTTTCATA	pSR47S
SacI_ravN_down_rev	AAAAGAGCTCGTGCATCGCCAGCACTTCTGCAT	pSR47S
ΔravN_for	GTAATACAGGTTGGGTTTAATGTGAAGTTGGCATAATAATAGTCCTTTAGC	pSR47S
ΔravN_rev	GCTAAAGGACTATTATTATGCCAACTTCACATTAAACCCAACCTGTATTAC	pSR47S
BglII_ravN_for	AAAGATCTCCAACTTATTTGATCCTATTAT	pGEX-6p-1
SalI_ravN_rev	AAGTCGACTTAATGTGAGTGTCCATGAGTA	pGEX-6p-1
HindIII_FLAG_TEV_ravN_for_a	GAAAACCTGTATTTTCAGGGCGGCAGCGGCCCAACTTATTTGATCCTATTAT	pcDNA5/FRO/TO
HindIII_FLAG_TEV_ravN_for_b	AAAAGCTTGCCATG*GACTACAAGGACGACGATGACAAA*GGCAGCGGCGAAAACCTGTATTTTCAGGGC	pcDNA5/FRO/TO
XhoI_ravN_rev	AACTCGAGTTAATGTGAGTGTCCATGAGTA	pcDNA5/FRO/TO
GST-ravN-BamHI_for	TCCGGAGGATCCATGCCAACTTATTTGATCCTATTATGCAAGAAGATAC	Crystallization
GST-ravN-XhoI_rev	TCCGGACTCGAGTCAATGTGAGTGTCCATGAGTATGCTCGC	Crystallization
GST-ravN-XhoI-123_rev	TCCGGACTCGAGTCATTGATCCTGTAGTACCTCTCGTGCCC	Crystallization
RavN_100_stop_BglII_for	GATAAGACTGAACCC**TAA**AGATCTAAGCAATTTAGTGAC	SD
RavN_100_stop_BglII _rev	GTCACTAAATTGCTTAGATCT**TTA**GGGTTCAGTCTTATC	SD
RavN_140_stop_BglII _for	TATCAACCCCTATTC**TAA**AGATCTATCCATGGGCATCAT	SD
RavN_140_stop_BglII _rev	ATGATGCCCATGGATAGATCT**TTA**GAATAGGGGTTGATA	SD
RavN_110_stop_BglII _for	TCCGTTAAGAGAGAG**TAA**AGATCTTTAACTGAAAACTGG	SD
RavN_110_stop_BglII _rev	CCAGTTTTCAGTTAAAGATCTTTACTCTCTCTTAACGGA	SD
RavN_120_stop_BglII _for	GCACGAGAGGTACTA**TAA**AGATCTCAGGATCAATTTGCT	SD
RavN_120_stop_BglII _rev	AGCAAATTGATCCTGAGATCT**TTA**TAGTACCTCTCGTGC	SD
RavN_123_stop_BglII _for	GTACTACAGGATCAA**TAA**AGATCTTTTGCTCATGCAGGG	SD
RavN_123_stop_BglII _rev	CCCTGCATGAGCAAAAGATCT**TTA**TTGATCCTGTAGTAC	SD
RavN_130_stop_BglII _for	CATGCAGGGCAAAAC**TAA**AGATCTCCTCATTTCTTTTTT	SD
RavN_130_stop_BglII _rev	AAAAAAGAAATGAGGAGATCT**TTA**GTTTTGCCCTGCATG	SD
RavN(I8S)_for	CTTATTTTGATCCT**AGT**ATGCAAGAAGATAC	SD
RavN(I8S)_rev	GTATCTTCTTGCAT**ACT**AGGATCAAAATAAG	SD
RavN(L43S)_for	GGATTGGAGCAC**AGC**CCCAGTGATCCAAC	SD
RavN(L43S)_rev	GTTGGATCACTGGG**GCT**GTGCTCCAATCC	SD
RavN(P47S)_for	CTCCCCAGTGAT**TCA**ACTACTCACGCGGAG	SD
RavN(P47S)_rev	CTCCGCGTGAGTAGT**TGA**ATCACTGGGGAG	SD
RavN(L43S/P47S)_for	GGATTGGAGCAC**AGC**CCCAGTGAT**TCA**ACTACTCACGCGGAG	SD
RavN(L43S/P47S)_rev	CTCCGCGTGAGTAGT**TGA**ATCACTGGG**GCT**GTGCTCCAATCC	SD
RavN(K101A)_for	AGACTGAACCC**GC**GCAATTTAGTGAC	SD
RavN(K101A)_rev	GTCACTAAATTGC**GC**GGGTTCAGTCT	SD
RavN(Q102A)_for	CTGAACCCAAG**GC**ATTTAGTGAC	SD
RavN(Q102A)_rev	GTCACTAAAT**GC**CTTGGGTTCAG	SD
RavN(F103A)_for	CTGAACCCAAGCAA**GC**TAGTGACTCCG	SD
RavN(F103A)_rev	CGGAGTCACTA**GC**TTGCTTGGGTTCAG	SD
RavN(S104A)_for	CCCAAGCAATTT**GC**TGACTCCGTTAAG	SD
RavN(S104A)_rev	CTTAACGGAGTCA**GC**AAATTGCTTGGG	SD
RavN(D105A)_for	CCCAAGCAATTTAGTG**C**CTCCGTTAAG	SD
RavN(D105A)_rev	CTTAACGGAG**G**CACTAAATTGCTTGGG	SD
RavN(S106A)_for	CCCAAGCAATTTAGTGAC**G**CCGTTAAG	SD
RavN(S106A)_rev	CTTAACGG**C**GTCACTAAATTGCTTGGG	SD
RavN(V107A)_for	GCAATTTAGTGACTCCG**C**TAAGAGAGAG	SD
RavN(V107A)_rev	CTCTCTCTTA**G**CGGAGTCACTAAATTGC	SD
RavN(K108A)_for	GCAATTTAGTGACTCCGTT**GC**GAGAGAG	SD
RavN(K108A)_rev	CTCTCTC**GC**AACGGAGTCACTAAATTGC	SD
RavN(R109A)_for	GTGACTCCGTTAAG**GC**AGAGTTAACTG	SD
RavN(R109A)_rev	CAGTTAACTCT**GC**CTTAACGGAGTCAC	SD
RavN(E110A)_for	GTGACTCCGTTAAGAGAG**C**GTTAACTG	SD
RavN(E110A)_rev	CAGTTAAC**G**CTCTCTTAACGGAGTCAC	SD
BamHI_AG_RavN_for	AAAAGGATCCAGATGCCAACTTATTTGATCCTATTATGC	pYES2/NTA

XbaI_RavN_rev	TTTTTCTAGATTAATGTGAGTGTCCATGAGTATGCTCG	pYES2/NTA
5'P_EcoRV_FLAG_PspXI_sense	ATCCTCGAGACCATG*GATTACAAGGATGACGATGACAAG*GGC	pcDNA6.2-FLAG
5'P_EcoRV_FLAG_PspXI_anti	TCGAGCC*CTTGTCATCGTCATCCTTGTAATC*CATGGTCTCGAGGAT	pcDNA6.2-FLAG
Lpg2452_C57A_for	GGCGATAATACG**GCC**AAATCGGATTTAGAAAG	SD
Lpg2452_C57A_rev	CTTTCTAAATCCGATTT**GGC**CGTATTATCGCC	SD
Lpg2452_H426A_for	GATGCTGAA**GCT**TTTGATGAACTAAGAATAG	SD
Lpg2452_H426A_rev	CTATTCTTAGTTCATCAAA**AGC**TTCAGCATC	SD
Lpg2452_D428A_for	GATGCTGAACATTTT**GCT**GAACTAAGAATAG	SD
Lpg2452_D428A_rev	CTATTCTTAGTTC**AGC**AAAATGTTCAGCATC	SD

SD, site-directed mutagenesis
