# Supplementary material for: RavN is a member of a previously unrecognized group of Legionella pneumophila E3 ubiquitin ligases
Source: PLoS Pathog. 2018 Feb 7;14(2):e1006897. doi: 10.1371/journal.ppat.1006897 (PMC5819833; doi:10.1371/journal.ppat.1006897)
Supplement: S1 Methods — (PDF) [file ppat.1006897.s012.pdf]

Supporting methods

***Legionella* growth in macrophages** - In-frame deletion of *ravN* from the genome of *L. pneumophila* strain Lp02 was generated as described in [1]. Intracellular growth assays were performed as described in [2]. Briefly, human U937 macrophage monolayers were challenged with the indicated *L. pneumophila* strains for 2 hours at a multiplicity of infection (MOI) of 0.05. After 2 hours, the monolayers were washed with PBS to remove extracellular bacteria, and covered with 500 μL RPMI medium. At 2, 24, 48, and 72 hours post-infection, digitonin (0.02% final concentration) was added to each well, and macrophage membranes were ruptured by shearing stress during pipetting. The supernatant was collected, the wells were rinsed with 500 μL PBS (0.02% digitonin), and the solution was combined with the initial lysate and then vigorously mixed by vortexing in order to disrupt residual membranes. Serial dilutions of the resulting cell lysate were spotted on CYET plates, incubated at 37 ℃ for three to four days, and colony-forming units (CFU) were determined for each dilution.

Fluorescence microscopy - Full-length *ravN* was cloned into pcDNA6.2/N-EmGFP-DEST via Gateway cloning (Life Technologies). Semi-confluent COS-1 cells were transiently transfected with pcDNA6.2/N-EmGFP-*ravN* or GFP vector control in 24-well plates using Lipofectamine 2000 (Life Technologies) and incubated overnight. Cells were chemically fixed with 3.8% formaldehyde in PBS, permeabilized by incubating with ice-cold methanol for 15 seconds, and blocked with 5% goat serum. Cell nuclei were stained with Hoechst, and coverslips were mounted in ProLong® Gold antifade reagent (Life Technologies) and imaged with a Zeiss Axio Observer.Z1 inverted light microscope.

Subcellular fractionation - U937 macrophages were seeded at a density of 3×10^6 cells per well in a 6-well plate and treated with 12.5 ng/mL 12-O-Tetradecanoylphorbol 13-acetate (TPA) for 48 hours. The medium was replaced with fresh RPMI and cells were incubated for another 24 hours before being challenged with Lp02 grown to post-exponential phase at an MOI of 100. After a two-hour infection period, the cells were harvested in PBS with protease inhibitors, and mechanically lysed in a dounce homogenizer. Cell debris, unbroken cells, and bacteria were pelleted by centrifugation at 15,000 rcf for 10 min at 4 ℃, and the supernatant (post-nuclear supernatant, PNS) was further centrifuged at 160,000 rcf for one hour at 4 ℃ to separate membrane components from the cytosol. The final supernatant was collected as the cytosolic fraction, and the pellet was resuspended with an equal volume of 2% Triton X-100 in PBS and collected as the membrane fraction. SDS sample buffer was added to each fraction and the original PNS, proteins were separated by SDS-PAGE, and analyzed by immunoblot.

Size-Exclusion Chromatography Coupled to Multiangle Light Scattering (SEC-MALS)

SEC-MALS was performed at room temperature using an ÄKTA Purifier (GE Healthcare) coupled with a DAWN HELEOS II MALS detector (Wyatt Technology) and an Optilab T-rEX differential refractometer (Wyatt Technology). 100 µL $RavN_{1\text{-}123}$ (2.5 mg/ml) were injected at 0.5 mL/min onto a Superdex 200 10/300GL column in a buffer containing 50 mM Tris-HCl pH 7.4, 150 mM NaCl and 10 mM BME. Light scattering and refractive index data were collected and analyzed using the ASTRA software package (Wyatt Technology). Bovine serum albumin was used as the calibration standard.

Circular Dichroism analysis - GST-tagged RavN or mutants were produced as described in Materials and Methods. After binding GST-tagged proteins onto Glutathione Sepharose resin, Precision Protease (GE Healthcare) was added directly to the resin slurry, and the resin slurry was incubated at 4 ℃ overnight. After on-resin cleavage by Precision Protease, untagged RavN was eluted and concentrated by Amicon Ultra Centrifugal Filters (Sigma) in PBS. Proteins were adjusted to a concentration of 0.2 mg/mL, and circular dichroism spectra were taken using a 1mm quartz cuvette in a Jasco J-715 spectrometer.

1. Luo Z-Q, Isberg RR. Multiple substrates of the Legionella pneumophila Dot/Icm system identified by interbacterial protein transfer. Proceedings of the National Academy of Sciences of the United States of America. 2004;101(3):841-6. doi: 10.1073/pnas.0304916101.
2. Conover GM, Derré I, Vogel JP, Isberg RR. The Legionella pneumophila LidA protein: a translocated substrate of the Dot/Icm system associated with maintenance of bacterial integrity. Molecular Microbiology. 2003;48(2):305-21. doi: 10.1046/j.1365-2958.2003.03400.x.
